# Effects of early tooth loss on chronic stress and progression of neuropathogenesis of Alzheimer’s disease in adult Alzheimer’s model *App^NL-G-F^* mice

**DOI:** 10.3389/fnagi.2024.1361847

**Published:** 2024-02-26

**Authors:** Suzuko Ochi, Kumiko Yamada, Takashi Saito, Takaomi C. Saido, Mitsuo Iinuma, Kagaku Azuma, Kin-Ya Kubo

**Affiliations:** ^1^Department of Pediatric Dentistry, Asahi University School of Dentistry, Mizuho, Japan; ^2^Department of Health and Nutrition, Faculty of Health Science, Nagoya Women’s University, Nagoya, Japan; ^3^Department of Neurocognitive Science, Institute of Brain Science, Nagoya City University Graduate School of Medical Sciences, Nagoya, Japan; ^4^Laboratory for Proteolytic Neuroscience, RIKEN Center for Brain Science, Wako, Japan; ^5^Department of Anatomy, School of Medicine, University of Occupational and Environmental Health, Kitakyushu, Japan; ^6^Graduate School of Human Life Science, Nagoya Women’s University, Nagoya, Japan

**Keywords:** Alzheimer’s disease, tooth loss, amyloid-β, phosphorylated tau, microglia, astrocyte

## Abstract

**Introduction:**

Alzheimer’s disease (AD), the most common neurodegenerative disease, is characterized by accumulated amyloid-β (Aβ) plaques, aggregated phosphorylated tau protein, gliosis-associated neuroinflammation, synaptic dysfunction, and cognitive impairment. Many cohort studies indicate that tooth loss is a risk factor for AD. The detailed mechanisms underlying the association between AD and tooth loss, however, are not yet fully understood.

**Methods:**

We explored the involvement of early tooth loss in the neuropathogenesis of the adult *App^NL-G-F^* mouse AD model. The maxillary molars were extracted bilaterally in 1-month-old male mice soon after tooth eruption.

**Results:**

Plasma corticosterone levels were increased and spatial learning memory was impaired in these mice at 6 months of age. The cerebral cortex and hippocampus of AD mice with extracted teeth showed an increased accumulation of Aβ plaques and phosphorylated tau proteins, and increased secretion of the proinflammatory cytokines, including interleukin 1β (IL-1β) and tumor necrosis factor α (TNF-α), accompanied by an increased number of microglia and astrocytes, and decreased synaptophysin expression. AD mice with extracted teeth also had a shorter lifespan than the control mice.

**Discussion:**

These findings revealed that long-term tooth loss is a chronic stressor, activating the recruitment of microglia and astrocytes; exacerbating neuroinflammation, Aβ deposition, phosphorylated tau accumulation, and synaptic dysfunction; and leading to spatial learning and memory impairments in AD model mice.

## 1 Introduction

With the rapid aging of society, the number of people with dementia is likely to increase. Dementia in aging populations is one of the largest health and economic issues globally. Japan is projected to have the world’s most aged population through 2050 (42.0% of the population over 65 years old) (United Nations, 2017). A 2018 study reported that the average prevalence of dementia in people 65 years and older is 15.8%, with Alzheimer’s disease (AD) being the most common type of dementia, accounting for 65.8% of all dementia cases (Montgomery et al., 2017). Global studies project that the fastest increasing prevalence of AD will occur in Japan, with AD cases increasing 4% annually between 2016 and 2026 (Global data, 2020). According to the 2021 World Alzheimer Report, approximately 55 million people have dementia worldwide. The number of individuals with dementia is predicted to increase to 78 million by 2030 and 139 million by 2050 (World Alzheimer Report, 2021). Dementia can be caused by many different diseases, and AD is the most common form (Selkoe, 2001; Wirths and Bayer, 2012).

Alzheimer’s disease is a severe neurodegenerative disease, characterized by progressive memory impairment and cognitive dysfunction. The main pathologic hallmarks of AD include the accumulation of extracellular amyloid-β (Aβ) plaques and aggregates of intracellular phosphorylated tau-laden neurofibrillary tangles, neuroinflammation, and synaptic and neuronal losses (Selkoe, 2001; Wirths and Bayer, 2012). Identifying the mechanisms underlying the cognitive impairment induced by AD is crucial to developing treatments and prevention. To this end, predictive AD model animals reflecting the disease progression of AD are essential. The first-generation AD mouse model was created based on the overexpression of amyloid precursor protein (APP) in the brain, with additional phenotypes that are not related to AD (Sasaguri et al., 2017). To overcome the problem of APP overexpression, Saito et al. developed a second-generation AD mouse model that incorporates humanized Aβ, *App^NL-G-F^* mice. In this mouse model, Aβ deposition begins at 2 months and cognitive impairment appears at 6 months.

This model exhibits pathophysiologic alterations induced by Aβ accumulation and avoids confusion caused by non-physiologic signals (Saito et al., 2014).

In the brains of people with AD and transgenic mouse models that overexpress mutated human APP, reactive microglia and astrocytes are observed around Aβ plaques and can have beneficial or harmful roles in disease progression (Dickson et al., 1988; Saito et al., 2014). Reactive microglia and astrocytes can contribute to the clearance of Aβ (Wyss-Coray et al., 2003; Mandrekar et al., 2009). Conversely, neuroinflammatory cytokines such as tumor necrosis factor (TNF-α), interleukin-6 (IL-6), and IL-1β produced following glial activation are harmful and toxic to neurons (Akiyama et al., 2000; Calsolaro and Edison, 2016). APP processing and Aβ accumulation result in the activation of microglia and astrocytes (Meraz-Rios et al., 2013). Inflammation occurs before the accumulation of the Aβ protein and plaque deposition are observed in APP transgenic mice (Wright et al., 2013), suggesting that inflammation is an early response in this AD model.

Poor oral health contributes to cognitive impairment (Kim et al., 2007; Stewart and Hirani, 2007; Naorungroj et al., 2013; Zenthöfer et al., 2014; Tsakos et al., 2015; Li et al., 2017). Cross-sectional studies reveal an association between cognitive impairment and poor oral health, including missing teeth (Kim et al., 2007; Stewart and Hirani, 2007). Cohort studies suggest that cognitive decline can result in poor oral health such as dental plaque accumulation (Zenthöfer et al., 2014), periodontal disease progression (Zenthöfer et al., 2014), and tooth loss (Naorungroj et al., 2013). Moreover, cohort studies suggest that tooth loss is a risk factor for cognitive decline (Tsakos et al., 2015; Li et al., 2017).

Animal studies using senescence-accelerated mouse prone 8 (SAMP8) indicate that tooth loss induces morphologic changes in the hippocampus, such as neuron loss, spine loss, impaired cell proliferation and synapse formation, glial cell changes, and fos induction, resulting in cognitive impairment (Onozuka et al., 1999; Watanabe et al., 2002; Kubo et al., 2005, 2014, 2021; Azuma et al., 2017; Katano et al., 2020). Tooth loss also induces increase in circulating corticosterone levels, indicating that tooth loss acts as a chronic stressor (Watanabe et al., 2002; Kubo et al., 2014). Several types of AD mouse models have been used to determine the effects of tooth loss on the development of AD (Oue et al., 2013, 2016; Goto et al., 2020; Taslima et al., 2021). For example, Goto et al. demonstrated that tooth extraction in *App^NL-G-F^* mice can trigger the spread of neurodegeneration from the mesencephalic trigeminal nucleus, locus coeruleus (LC), and hippocampus, thereby accelerating the onset of AD (Goto et al., 2020). To date, it remains unclear, however, whether tooth loss promotes the development of AD via the amyloid cascade in AD model mice (Oue et al., 2013, 2016; Taslima et al., 2021). In the present study, we explored the effects of tooth extraction soon after tooth eruption on the neuropathogenesis of *App^NL-G-F^* AD model mice to examine the role of oral health in maintaining cognitive function.

## 2 Materials and methods

### 2.1 Animal and experimental protocol

The *App^NL-G-F^* mice (male, 1-month of age) used in the present study were provided free of charge by the RIKEN Center for Brain Science (Saito et al., 2014). Experimental protocols were approved by the ethics committee for animal care and experimentation at Asahi University School of Dentistry (permission code: 20–024). The mice were maintained on standard pellet chow (CE-2, CLEA Japan, Inc., Tokyo, Japan) and drinking water *ad libitum*. Mice were housed under temperature (22 ± 2°C), humidity (55 ± 2%), and light/dark cycle (lights on at 6:00–18:00) controlled conditions. As mouse tooth development contains for 25 days after birth (Hamajima, 1963), the maxillary molar teeth were removed bilaterally when the mice were 1 month of age.

Mice were injected intraperitoneally with an anesthetic mixture (0.1 ml/10 g body weight) comprising medetomidine 0.75 ml, midazolam 2.0 ml, and butorphanol 2.5 ml diluted in 19.75 ml distilled water. The maxillary molar teeth were extracted using dental tweezers. Control mice underwent the same procedures without extraction of the molar teeth. After extraction, all animals were maintained under standard conditions for 5 months. Aβ plaque deposition begins at 2 months and cognitive impairment appears at 6 months of age in *App^NL − G − F^* mice (Saito et al., 2014; Masuda et al., 2016). We examined the effects of tooth loss on learning ability and brain histology (hippocampus and cerebral cortex) at 6 months of age when learning impairment started and before peak of Aβ plaque deposition. To evaluate the mean lifespan, the mice were housed under normal conditions until they died naturally. The mean longevity of each group was recorded (*n* = 28/group).

### 2.2 Morris water maze test

The Morris water maze test was conducted for experimental and control animals (n = 7/group) as indicated previously (Kubo et al., 2014). A circular stainless steel pool (diameter: 90 cm × 30 cm tall) was filled with warm water (temperature: 28°C) to a height of 23 cm. A platform was submerged 1 cm under the water surface. Animals were placed in the water from 1 of 4 randomly assigned locations and allowed to find and escape to the submerged platform. All animals were given 4 trials daily for 7 consecutive days. A CCD camera (Exwane, Sony, Tokyo, Japan) linked to a computer system (Movetr/2D, Library Co. Ltd., Tokyo, Japan) was used to record the latency to swim to and mount the platform. On the last trial day, to rule out visuomotor or motivation impairment, a probe test was performed in which the platform was visible above the water surface.

### 2.3 Plasma corticosterone levels

To investigate the effects of tooth loss on changes in the circulating stress hormone levels in *App^NL − G − F^* mice, we measured the plasma corticosterone levels in 6-month-old mice (*n* = 10/group). Mice were injected intraperitoneally with the anesthetic mixture used for the tooth extraction surgery.

Blood samples were collected from the heart and centrifuged at 3500 rpm for 10 min at 4°C to separate the plasma. The plasma corticosterone levels were determined using an enzyme-linked immunosorbent assay kit according to the manufacturer’s instructions (Assaypro, St. Charles, MO, USA).

### 2.4 Immunofluorescence staining

At 6 months of age, mice were injected intraperitoneally with the anesthetic mixture used for the tooth extraction surgery. The mice were transcardially perfused with 0.9% sodium chloride, followed by 4% paraformaldehyde in 0.1 M phosphate buffer at pH 7.4 through the ascending aorta. The brains were removed and immersed in the same fixative for 24 h at 4°C, followed by gradient ethanol dehydration. Paraffin sections (4-µm thick) were prepared and treated with xylene and ethanol, followed by antigen retrieval using an autoclave at 121°C for 5 min. Sections were incubated in blocking solution for 10 min and then with anti-human Aβ (N; 82E1, 1:100, Immuno-Biological Laboratories Co., Ltd., Gunma, Japan) overnight at 4°C. After washing with phosphate-buffered saline (PBS), the sections were incubated with streptavidin-HRP (1:500, PerkinElmer, Boston, MA, USA) for 30 min and then treated with the TSATM Coumarin System (1:100, PerkinElmer) for 10 min. After rinsing with PBS, the sections were mounted using aqua-polymount (PSI, Warrington, PA, USA). The percent area of the immunoreactive Aβ plaque in the hippocampus and cortex (*n* = 6/group) was calculated using Image J (NIH, Rockville, MD, USA) as previously described (Schneider et al., 2012). The primary antibodies used in this study are summarized in Table 1.

**Table 1:** Antibodies used in this study.

Antibody	CAT#	Source	Dilution	Application
Amyloid-β	#10326	IBL	1:100	IF
Iba1	#19–19741	FUJIFILM	1:500	IF
GFAP	#MAB360	Millipore	1:500	IF
Synaptophysin	#ab52636	Abcam	1:1000	WB
Phospho-tau	#90343	Fujirebio	1:1000	WB
β-Actin	#4967S	CST	1:1000	WB

CST, Cell signaling technology; IBL, Immuno-Biological Laboratories Co., Ltd.; IF, immunofluorescence; WB, Western blot.

For immunohistochemical evaluation of microglia and astrocytes, deparaffinized sections were treated with citrate buffer containing 0.1% Tween 20 for antigen retrieval. To inhibit endogenous peroxidase, the sections were soaked in methanol containing 0.3% H2O2. After washing with PBS, the sections were incubated with primary antibody, anti-Iba1 (1:500, Wako, Osaka, Japan) or anti-glial fibrillary acidic protein (GFAP, 1:100, clone GA5, EMD Millipore Corporation, Temecula, CA, USA) overnight at 4°C. After washing with PBS, the sections were incubated with goat anti-rabbit IgG H&L (1:500, Alexa Fluor^Ⓡ^568, Abcam, Carlsbad, CA, USA) and goat anti-mouse IgG H&L (1:500, Alexa Fluor^Ⓡ^488, Abcam) for 2 h. After washing in PBS, the sections were treated with tris-NaCl-blocking buffer for 30 min. The sections were then incubated with anti-human Aβ (N; 82E1, 1:100, ImmunoBiological Laboratories Co., Ltd) for 24 h at 4°C, treated with streptavidin-HRP (1:500, PerkinElmer) and the TSATM Coumarin System (1:100, PerkinElmer). The percent area of the Iba1-positive microglia and GFAP-positive astrocytes was determined using Image J (NIH, n = 6/group).

### 2.5 Western blot analyses of phosphorylated tau protein and synaptophysin

At 6 months of age, mice (*n* = 6/group for each protein) were anesthetized with the anesthetic mixture used for the molar extraction surgery and killed by cervical dislocation, the brains were quickly removed. The cerebral cortex and hippocampus were lysed with RIPA lysis buffer system (Santa Cruz Biotechnology Inc., Dallas, TX, USA) or RIPA lysis buffer system added to Phosphatase Inhibitor Cocktail Solution II (Wako, USA) and centrifuged at 10,000× g for 10 min at 4°C to collect the supernatant. The protein concentration was detected using a Pierce BCA Protein Assay Kit (Thermo Fisher Scientific, Waltham, MA, USA). The proteins were separated by sodium dodecyl sulfate polyacrylamide gel electrophoresis with a 10% polyacrylamide gel (ATTO Corp., Tokyo, Japan) and transferred to a polyvinylidene difluoride membranes (Millipore Corp., Billerica, MA, USA). After blocking with 0.5% casein (Milipore Sigma, St. Louis, MO, USA) or EzBlock Chemi (ATTO Corp.), immunoblotting was carried out using the primary antibodies, rabbit anti-synaptophysin (1:1000, Abcam), anti-phospho-tau (AT8) (1:1000, Fujirebio Europe N.V., Gent, Belgium), anti-β-Actin (1:1000, Cell Signaling Technology, Danvers, MA, USA) overnight at 4°C. The membranes were incubated with the secondary antibodies (1:1000, Cell Signaling Technology) for 30 min. After rinsing in tris-buffered saline, an Immobilon Western Chemiluminescent HRP Substrate (Millipore Corp.) was used to detect the bands of the target protein. Quantitative analysis was performed using ATTO Densitograph software library (ATTO Corp., Tokyo, Japan).

### 2.6 Real time polymerase chain reaction test

Following the induction of deep anesthesia with the anesthetic mixture used for the molar extraction surgery, the hippocampus and cerebral cortex were removed. RNA was then extracted using Directzol™ RNA MiniPrep w/Tri Reagent (Zymo Research Irvine, CA, USA), and cDNA was synthesized using Prime Script™ reverse transcription polymerase chain reaction (PCR) kit (TaKaRa, Shiga, Japan). Quantitative real time PCR was performed using the Thermal Cycler Dice R Real Time System Software (TaKaRa) with Premix Ex TaqTM (Perfect Real Time; Takara). The sequences of the primers used in this study are as follows:

IL-1β forward: 5’-CCAGGATGAGGACATGAGCAC-3′, reverse: 5’-TGTTGTTCATCTCGGGAGCCTGTA-3′; TNF-α forward: 5’-GCCTCTTCTCATTCCTGCTTGTG-3′, reverse: 5’-TGATGsAGAGAGGGAGGCCATTTG-3′; and β-actin forward: 5’-CATCCGTAAAGACCTCTCTATGCCA-3′, reverse: 5’-ATGGAGCCACCGATCCACA-3′.

IL-1β and TNF-α mRNA expression levels were normalized against β-actin expression levels. Relative mRNA expression levels were calculated using the 2-ΔΔCt method.

### 2.7 Statistical analyses

All experimental data are expressed as mean ± SEM. The variables of the Morris Water maze test were statistically analyzed using a two-way repeated measures analysis of variance. The other parameters were statistically compared using Student’s *t*-test. A *p-*value of less than 0.05 was considered statistically significant.

## 3 Results

### 3.1 Plasma corticosterone levels

**Figure 1:**
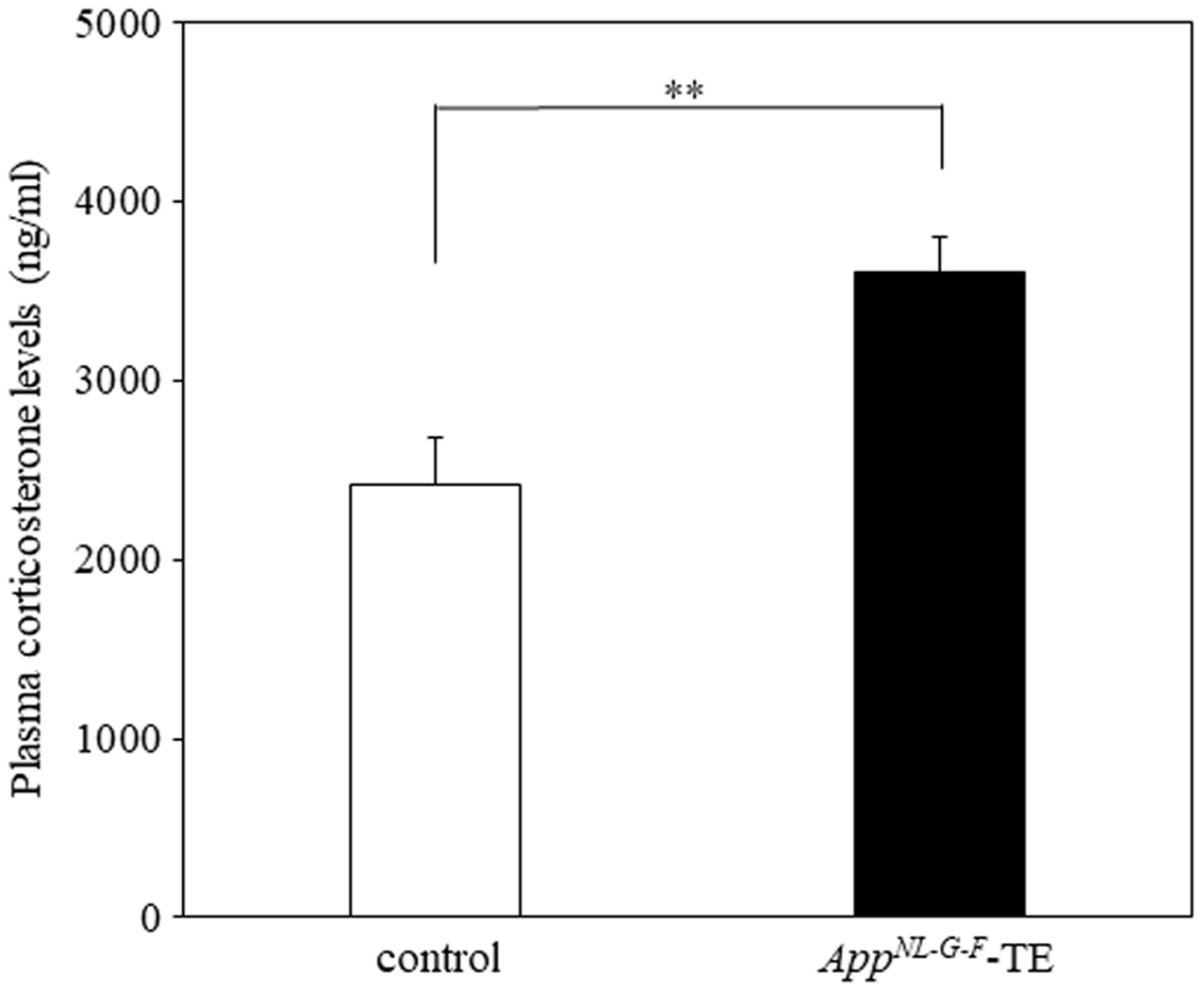
Plasma corticosterone levels in control mice and *App^NL-G-F^* mice with early tooth loss Compared with the control mice, plasma corticosterone levels were significantly higher in the *App^NLG-F^* mice with early tooth loss (*App^NL-G-F^*-TE; *p* < 0.01). Data are expressed as mean ± SEM. *n* = 10/group. **p < 0.01.

Plasma corticosterone levels were significantly higher in the *App^NL-G-F^* tooth extraction (*App^NL-G-F^* – TE) group than in the control group (2420.8 ± 80.1 vs. 3606.0 ± 61.4, *p* = 0.0007, Figure 1), indicating that permanent tooth loss induced chronic stress.

### 3.2 Morris water maze test

**Figure 2:**
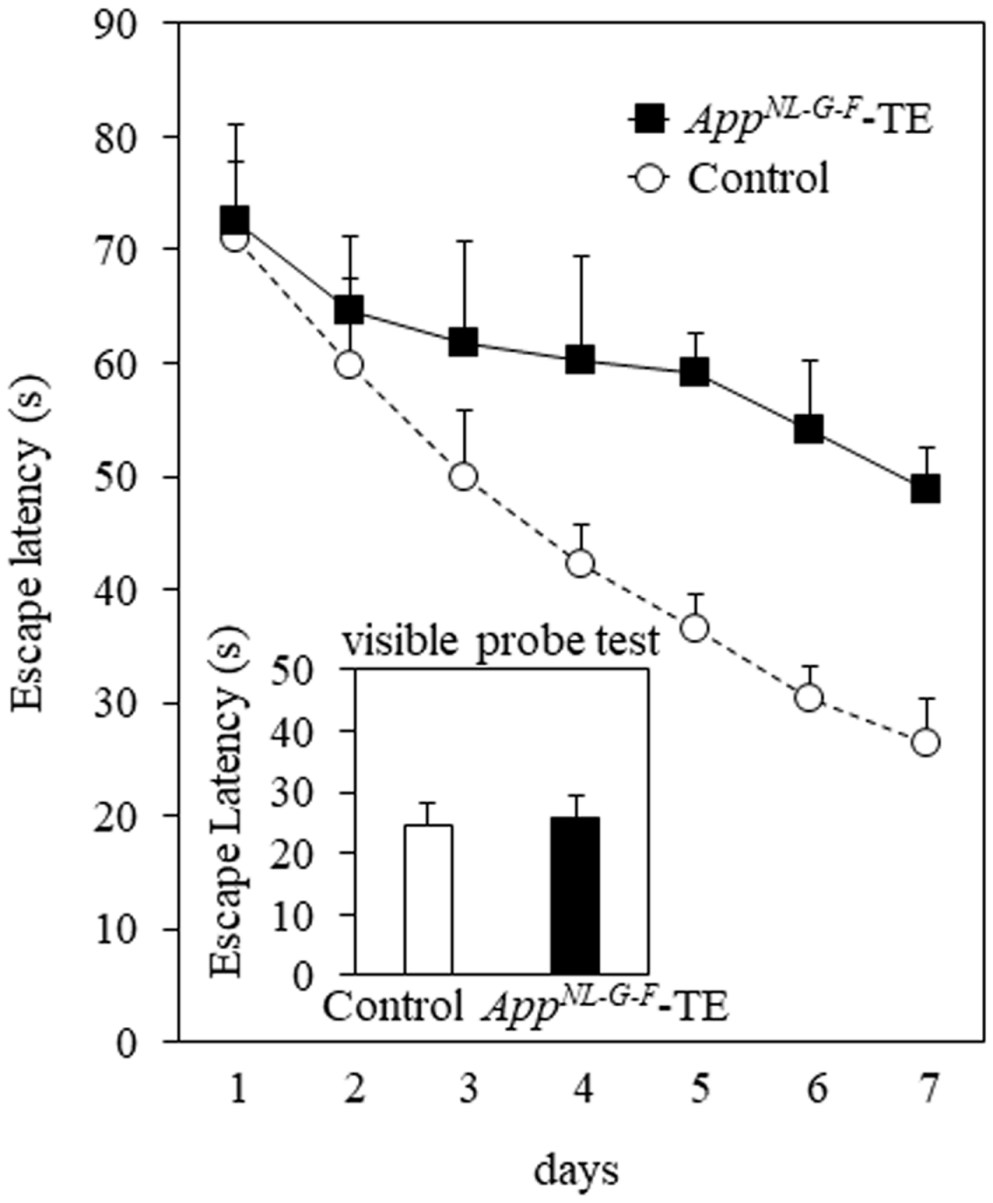
Spatial learning in the Morris water maze test The mean daily (mean of the 4 trials each day) escape latency was longer than in the *App^NL-G-F^* mice with early tooth loss (*App^NL-G-F^*-TE) than in the control mice (*p* < 0.01). No significant differences were detected between the 2 groups in the visible probe test, indicating similar visual and motor abilities in both groups. Data are expressed as mean ± SEM. *n* = 7/group.

Mice in both groups exhibited learning based on a decrease in the escape latency during the 7 days of training of the Morris water maze test. The mean daily escape latency was significantly longer in the *App^NL-G-F^*-TE group than in the control group (*p* < 0.01), indicating that tooth loss early in life further impairs spatial learning in *App^NL-G-F^* mice. No significant difference was detected between the 2 groups in the visible probe test (24.68 ± 3.66 vs. 25.61 ± 3.69, *p* = 0.861), indicating similar visual and motor abilities in the *App^NL-G-F^*-TE and control mice (Figure 2).

### 3.3 Amyloid β plaque

**Figure 3:**
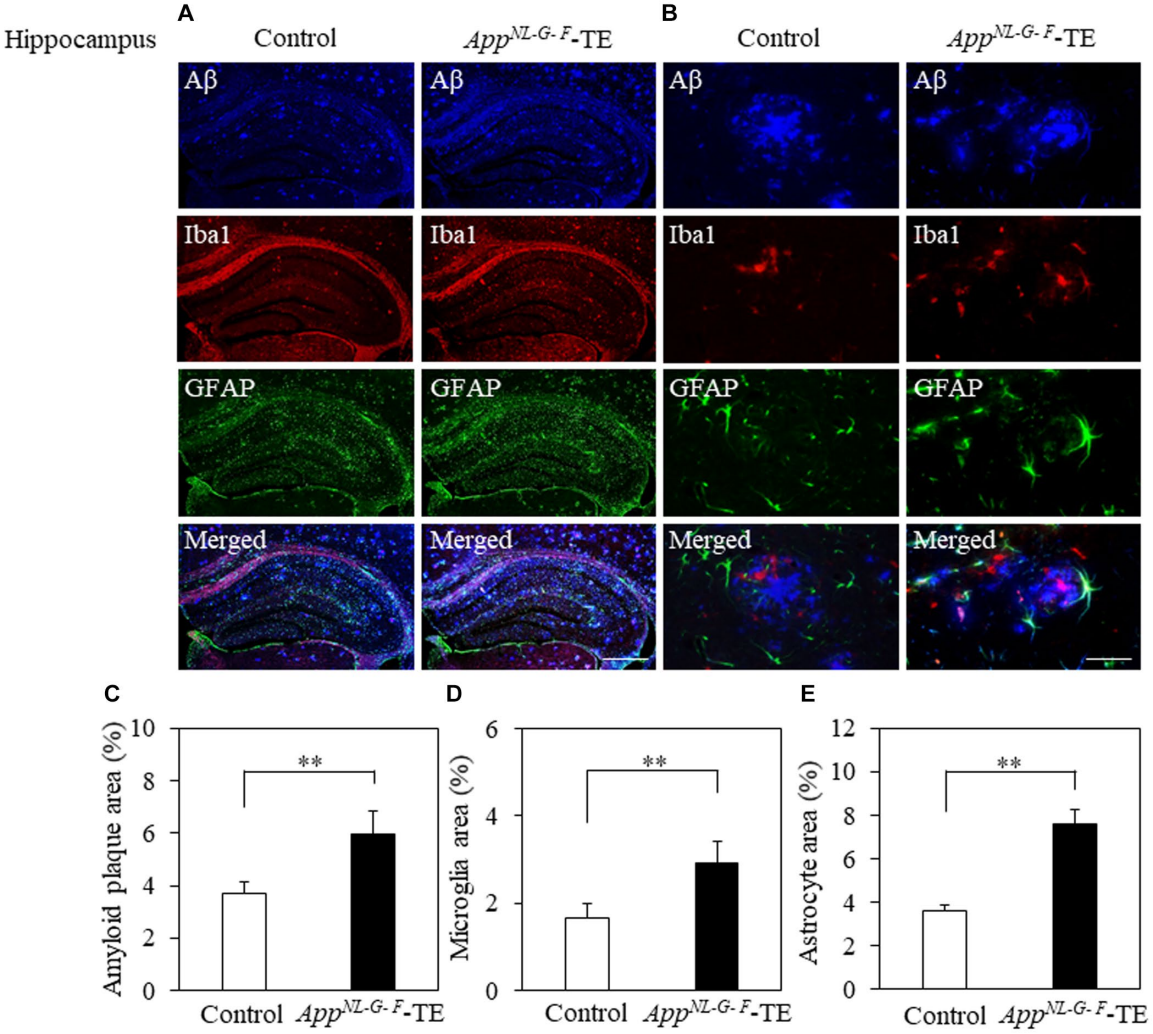
Aβ plaques, microglia, and astrocyte recruitment in the hippocampus Immunofluorescence images of Aβ plaques, Iba1-immunopositive cells, and GFAP-immunopositive cells in the hippocampus of the control mice and *App^NL-G-F^* mice with early tooth loss (*App^NL-G-F^*-TE). Increased Iba1-immunopositive cells and GFAP-immunopositive cells were observed around the Aβ plaques in the hippocampus of the *App^NL-G-F^*-TE mice, indicating widespread neuroinflammation in the hippocampus. As compared with control mice, the percentage of the Aβ plaque area in the hippocampus was significantly higher in the *App^NL-G-F^*-TE mice (**C**: *p* < 0.01). The percentage of area occupied by microglia (**D**: *p* < 0.01) and astrocytes (**E**: *p* < 0.01) was significantly higher in the *App^NLG-F^*-TE mice than in the control mice. **(A)** Low power field (x100), scale bar: 500 μm, **(B)** high power field (x400), scale bar: 40 μm. Data are expressed as mean ± SEM. *n* = 6/group. **p < 0.01.

**Figure 4:**
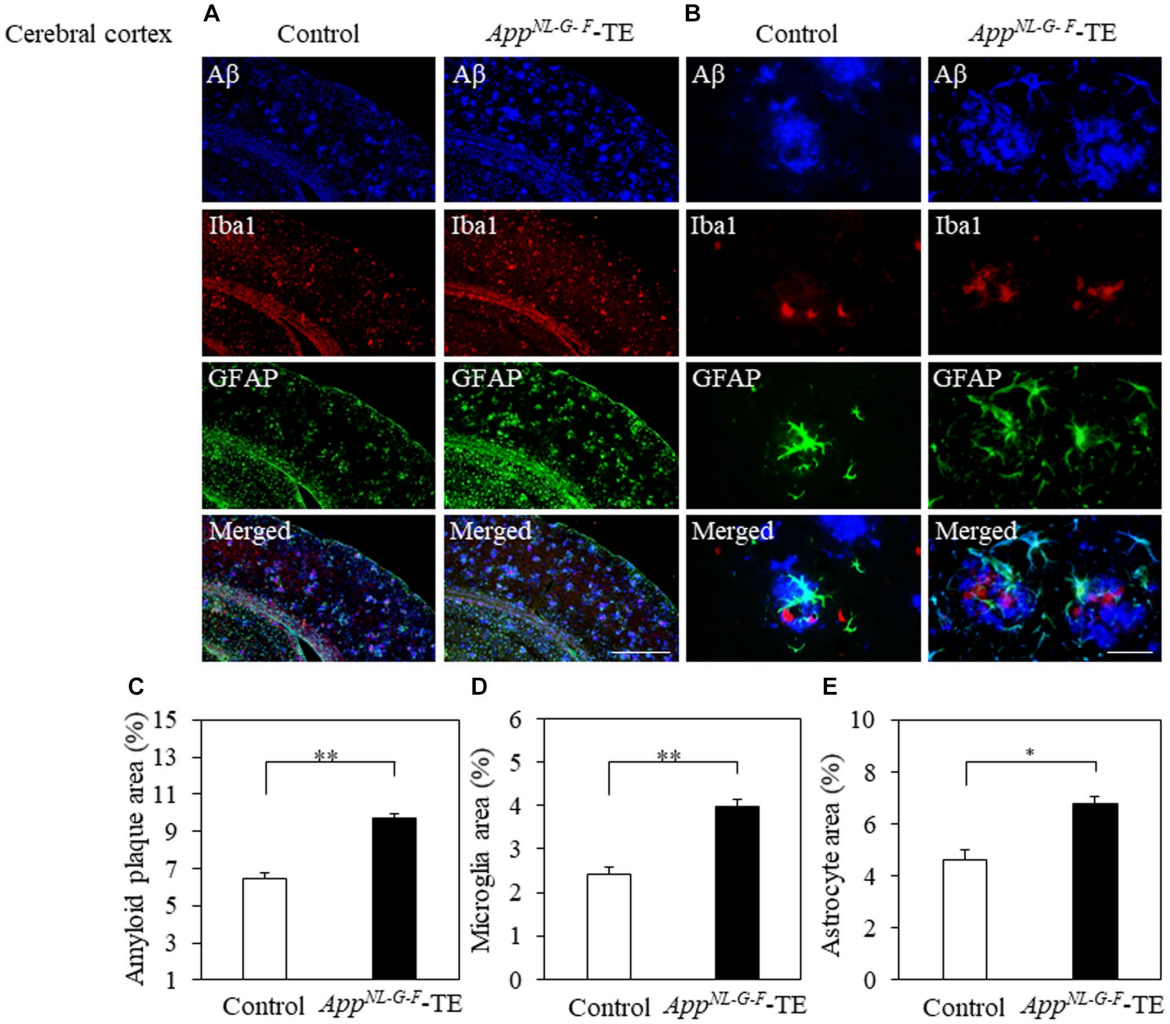
Aβ plaques, microglia and astrocyte recruitment in the cerebral cortex Immunofluorescence images of Aβ plaques, Iba1-immunopositive cells, GFAP-positive cells, and superimposed photos of Aβ plaques, Iba1-and GFAP-immunopositive cells in the cerebral cortex of the control mice and *App^NL-G-F^* mice with early tooth loss (*App^NL-G-F^*-TE). Increased Iba1immunopositive cells and GFAP-immunopositive cells were observed around the Aβ plaques in the cerebral cortex of *App^NL-G-F^*-TE mice, indicating widespread neuroinflammation in the cerebral cortex. As compared with the control mice, the percentage of the Aβ plaque area in the cerebral cortex was significantly higher in the *App^NL-G-F^*-TE mice (**C**: *p* < 0.01). The percentage of area occupied by microglia (**D**: *p* < 0.01) and astrocytes (**E**: *p* < 0.05) was significantly higher in the *App^NL-G-F^*-TE mice than in the control mice. **(A)** Low power field (x100), scale bar: 500 μm, **(B)** high power field (x400), scale bar: 40 μm. Data are expressed as mean ± SEM. *n* = 6/group. *p < 0.05, **p < 0.01.

Aβ deposition was detected in the hippocampus and cerebral cortex of both groups (Figures 3A,B, 4A,B). The percentage of Aβ plaque area in both the cerebral cortex (6.45 ± 0.296 vs. 9.72 ± 0.208, *p* = 0.0008) and hippocampus (3.66 ± 0.259 vs. 5.96 ± 0.364, *p* = 0.0006) was significantly greater in the *App^NL-G-F^*-TE group than in the control group (Figures 3C, 4C), indicating a remarkable increase in Aβ plaque in *App^NL-G-F^*-TE mice associated with early tooth loss.

### 3.4 Neuroinflammation

To determine the changes in the accumulation of microglia and astrocytes associated with Aβ plaque deposition, we performed triple immunofluorescence straining. Iba1-and GFAP-immunopositive cells were observed in the hippocampus and cerebral cortex of both groups (Figures 3A,B, 4A,B). The increased concentration of microglia and astrocytes around Aβ plaque is strongly indicative of neuroinflammation. The percent area occupied by Iba1-immunopositive cells and GFAP-immunopositive cells in the hippocampus (1.66 ± 0.133 vs. 2.92 ± 0.198, *p* = 0.0005; 3.69 ± 0.111 vs. 7.56 ± 0.283, *p* = 0.0007, respectively, Figures 3D,E) and cerebral cortex (2.41 ± 0.160 vs. 3.99 ± 0.151, *p* = 0.0003; 4.63 ± 0.391 vs. 6.78 ± 0.280, *p* = 0.0012, respectively, Figures 4D,E) was significantly greater in the *App^NL-G-F^*-TE group than in the control group, indicating gliosis in *App^NL-G-F^* mice associated with early tooth loss. Numerous fluorescently labeled Iba1-and GFAP-immunopositive cells concentrated around Aβ plaques in both the hippocampus and cerebral cortex (Figures 3B, 4B), indicating that neuroinflammation was induced around Aβ plaques by microglia and astrocytes.

### 3.5 Phosphorylated tau protein (AT8/β-actin)

**Figure 5:**
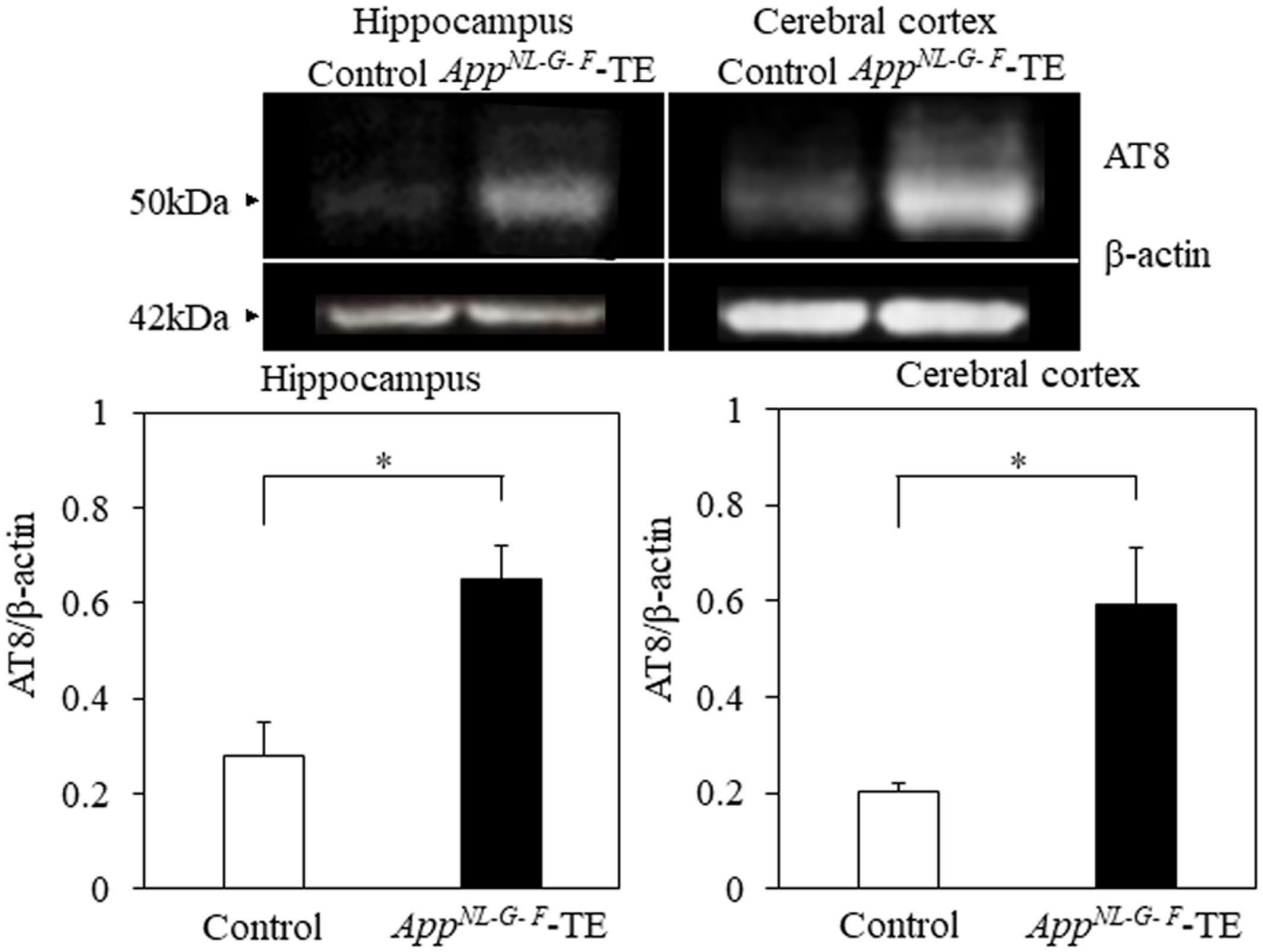
Expression levels of phosphorylated tau protein in the cortex and hippocampus Expression levels of phosphorylated tau protein in both the cerebral cortex (*p* < 0.05) and hippocampus (*p* < 0.05) were significantly higher in the *App^NL-G-F^* mice with early tooth loss than in the control mice. Data are expressed as mean ± SEM. *n* = 6/group. *p < 0.05.

Phosphorylated tau protein (AT8/β-actin) expression levels in the hippocampus (0.28 ± 0.069 vs. 0.65 ± 0.073, *p* = 0.004) and cortex (0.20 ± 0.018 vs. 0.59 ± 0.115, *p* = 0.007) were significantly increased in the *App^NL-G-F^*-TE group compared with the control group (Figure 5), indicating that increased phosphorylated tau protein in the *App^NL-G-F^*-TE mice was related to early tooth loss.

### 3.6 mRNA expression levels of IL-1β and TNF

**Figure 6:**
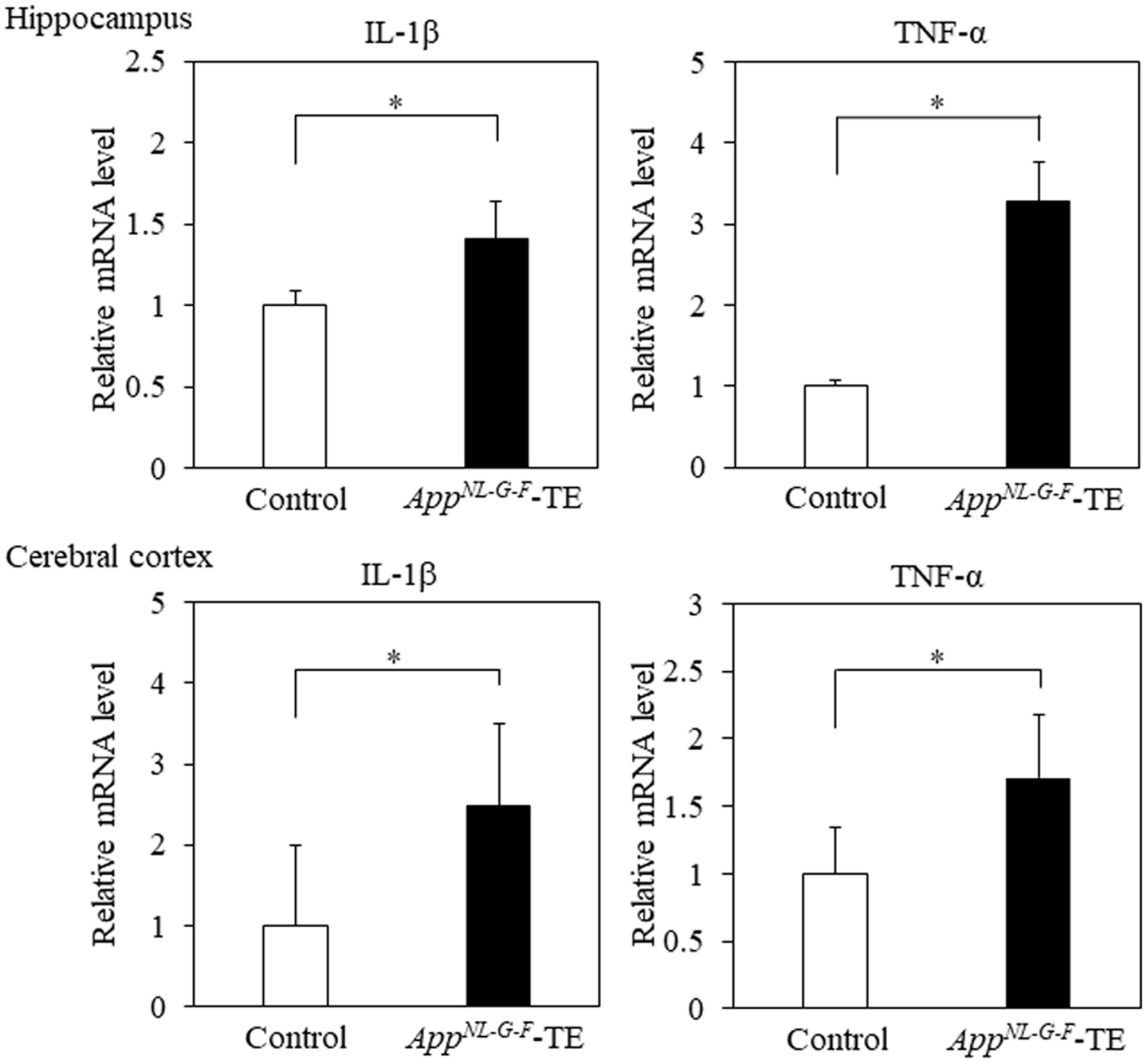
Expression levels of IL-1β and TNF-α in the cerebral cortex and hippocampus Expression levels of IL-1β in both the cerebral cortex (*p* < 0.05) and hippocampus (*p* < 0.05) were significantly higher in the *App^NL-G-F^* mice with early tooth loss (*App^NL-G-F^*-TE) than in the control mice. Expression levels of TNF-α both in the cerebral cortex (*p* < 0.05) and hippocampus (*p* < 0.05) were significantly higher in the *App^NL-G-F^*-TE mice than in the control mice. Data are expressed as mean ± SEM. *n* = 6/group. *p < 0.05.

The mRNA expression levels of IL-1β in the hippocampus (0.91 ± 0.085 vs. 1.28 ± 0.097, *p* = 0.016) and cerebral cortex (1.80 ± 0.203 vs. 4.47 ± 0.675, *p* = 0.009) were higher in the *App^NL-G-F^*-TE mice than in the control mice (Figure 6). The mRNA expression levels of TNF-α in the hippocampus (1.05 ± 0.084 vs. 3.28 ± 0.741, *p* = 0.014) and cerebral cortex (21.98 ± 4.396 vs. 37.68 ± 5.241, *p* = 0.0446) were also higher in the *App^NL-G-F^*TE mice than in the control mice (Figure 6).

### 3.7 Synaptophysin

**Figure 7:**
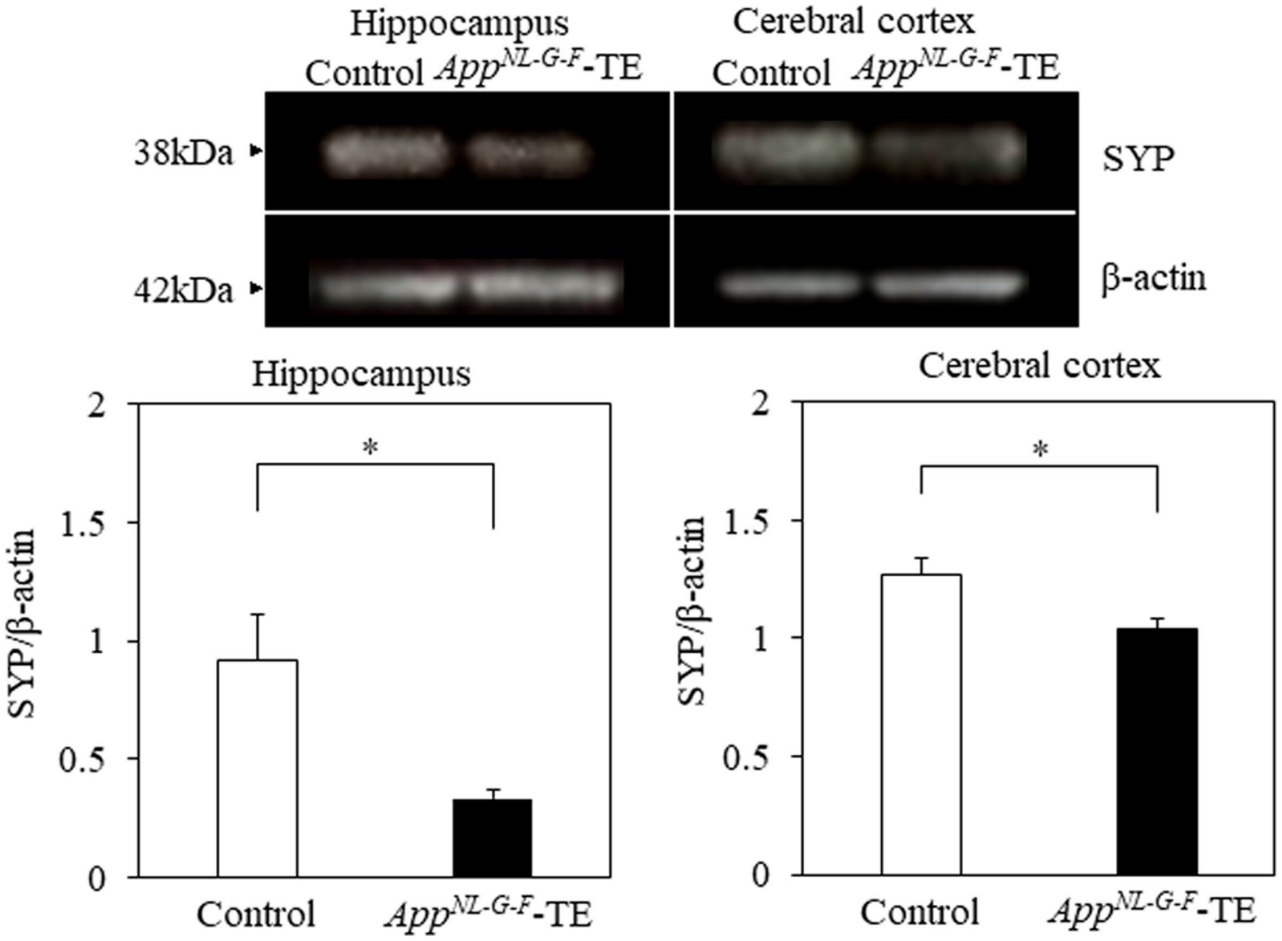
Expression levels of synaptophysin in the cerebral cortex and hippocampus Expression levels of synaptophysin in both the cerebral cortex (*p* < 0.05) and hippocampus (*p* < 0.05) were significantly lower in the *App^NL-G-F^* mice with early tooth loss (*App^NL-G-F^*-TE) than in the control mice. Data are expressed as mean ± SEM. *n* = 6/group. *p < 0.05.

Synaptophysin expression levels in both the hippocampus (0.99 ± 0.142 vs. 0.46 ± 0.044, *p* = 0.005) and cerebral cortex (1.27 ± 0.074 vs. 1.04 ± 0.040, *p* = 0.0238) were significantly lower in the *App^NL-G-F^*-TE mice than in the control mice (Figure 7), indicating synaptic dysfunction in the *App^NL-G-F^*-TE mice related to permanent tooth loss.

### 3.8 Lifespan

**Figure 8:**
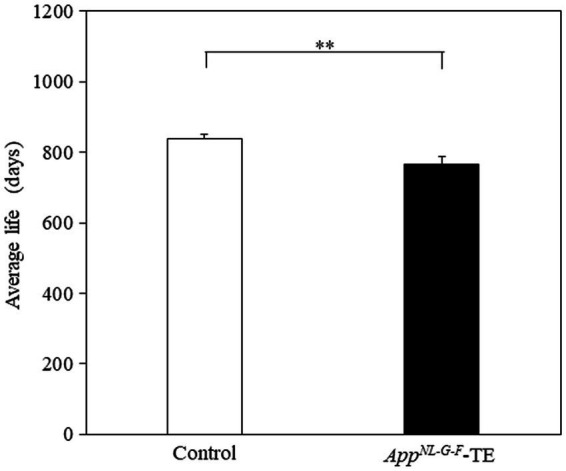
Average longevity of control mice and *App^NL-G-F^* mice with early tooth loss Survival time was significantly shorter in the *App^NL-G-F^* mice with early tooth loss (*App^NL-G-F^*-TE) than in the control mice (*p* < 0.01). Data are expressed as mean ± SEM. *n* = 28/group. **p < 0.01.

The mean lifespan of the control and *App^NL-G-F^*-TE groups was 839 and 767 days, respectively (Figure 8). The survival times were significantly shorter in the *App^NL-G-F^*-TE group than in the control (8.6%; 839.29 ± 12.05 vs. 766.61 ± 22.66, *p* = 0.0071).

## 4 Discussion

The major findings of the current study indicated that tooth loss early in life accelerated spatial learning impairments, increased accumulation of Aβ plaques and phosphorylated tau protein, increased the amount of microglia and astrocytes around Aβ plaques, increased the secretion of IL-1β and TNFα, and decreased synaptophysin expression in the cerebral cortex and hippocampus in *App^NL-G-F^* mice. Tooth loss early in life enhanced the spatial learning impairment observed in *App^NL-G-F^* mice, corresponding with the previous findings using AD model mice (Oue et al., 2013; Taslima et al., 2021) and SAMP8 mice (Onozuka et al., 1999; Watanabe et al., 2002; Kubo et al., 2005, 2014, 2021).

Overwhelming evidence indicates that an accumulation of Aβ plaques in the brain is an early pathologic feature of AD. Abnormal accumulation of Aβ plaques leads to the formation of senile plaques and impairs cognitive function (Gulisano et al., 2018; Zhang et al., 2021). Tau protein, mainly existing in neurons, stabilizes microtubules and is essential for neural transport. During senile plaque formation, tau phosphorylated by tau kinases, leading to the formation of tau aggregates, including highly phosphorylated paired helical filaments and neurofibrillary tangles, which are toxic to neurons and synapses (Gulisano et al., 2018; Zhang et al., 2021).

Microglia play a major role in the immune function of the central nervous system against various pathogens and external invasion factors, and remove damaged or dead neurons (Yang et al., 2010). Astrocytes are the major type of glial cell in the brain and play critical roles in maintaining the normal function of the brain, such as by regulating synaptogenesis, and supporting neurogenesis, and form a protective barrier function between Aβ and neurons (Frost and Li, 2017). Aβ activates microglia via the receptor for advanced glycation endproducts and activated microglia undergo amoeboid-like morphologic changes and are implicated in active phagocytosis of Aβ, facilitating its degradation and excretion (Cai et al., 2022). On the other hand, activated microglia also cause neuronal damage by releasing proinflammatory substances, including IL-1β, IL-6, TNF-α, and nitric oxide. Microglia are further activated to remove the damaged neurons, forming a vicious cycle that promotes neuroinflammation. These substances serve as messengers between neurons and glia. In the case of AD, they act as inflammatory triggers, contributing to neuroinflammation and promoting Aβ aggregation (Cai et al., 2022). In AD, Aβ neurogenic changes activate microglia and astrocytes, and induce the accumulation of activated microglia/astrocytes around senile plaque. Like microglia, activated astrocytes are involved in the degradation and excretion of Aβ. In the presence of Aβ, astrocytes are activated, inducible nitric oxide synthase is induced, and various proinflammatory substances are secreted. Furthermore, activated astrocytes may contribute to the pathogenesis of amyloid angiopathy by altering capillary function and the blood–brain barrier. Microglia and astrocytes play dual roles, protecting the brain and yet increasing neuroinflammation in the brain, and are thus both involved in the onset and exacerbation of AD (Kwon and Koh, 2020).

Synapses are specialized junctions designed to ensure efficient and accurate communication between neurons. Synaptic transmission relies on the fast, efficient, and synchronous release of chemical neurotransmitters. Loss of synapses is an early pathologic hallmark of various neurodegenerative diseases, including AD, and is strongly correlated with cognitive impairment (Terry et al., 1991). In AD, Aβ oligomers impair synaptic function, causing synaptic loss and cognitive impairment (Gulisano et al., 2018; Zhang et al., 2021). In experimental animals, synaptic loss is associated with tau hyperphosphorylation and memory impairment (Gulisano et al., 2018; Zhang et al., 2021).

Indeed, increased accumulation of Aβ surrounded by microglia and astrocytes, increased phosphorylated tau protein levels, and a pronounced decrease in spatial learning were observed in the *App^NL-G-F^*-TE mice. The mRNA expression levels of IL-1β and TNF-α were also increased and synaptophysin levels were decreased in the *App^NL-G-F^*-TE mice. These findings suggest that the histologic and functional changes observed in AD, including neuroinflammation and synaptic damage, are more advanced in *App^NL-G-F^* mice with early tooth loss than intact *App^NL-G-F^* mice. Therefore, presumably, tooth loss precipitates the progression of AD.

The progression of AD pathology due to tooth loss may be induced by the following 2 factors. One possibility is reduced information input into central nervous system or nerve degeneration, and the other possibility is chronic stress induced by tooth loss. Sensory receptors of the pulp and periodontal ligament are damaged owing to continuing dysfunction of mastication after tooth extraction, resulting in variations of their neuronal pathways. Tooth extraction leads to degenerative alterations in trigeminal ganglion cells and nerve fibers of the sensory neurons innervating the teeth (Gobel, 1984; Kubota et al., 1988). Tooth loss induces neural cell death in the trigeminal mesencephalic nucleus (Vmes) and neuronal degeneration in the trigeminal motor nucleus, affecting masticatory function (Dhar et al., 2021). Goto et al. reported Aβ was deposition in Vmes neurons of triple transgenic-AD mice after tooth extraction (Goto et al., 2020). The release of cytotoxic Aβ peptides due to the death of Vmes neurons triggered by tooth loss, causes damage to neurons adjacent to the Vmes in the lateral part of the periaqueductal gray matter of the fourth ventricle such as in the LC, and the LC and Vmes are interconnected (Takahashi et al., 2010; Goto et al., 2020). In addition, the LC is the first brain region in which AD develops (Mather and Harley, 2016), and the volume of the human LC decreases according to the degree of pathology in Parkinson’s disease and AD (Theofilas et al., 2017). The LC projects axons to many areas, including the cerebral cortex and hippocampus, and plays an important role as a central noradrenaline producer; damage to the LC directly affects hippocampal function (Kelly et al., 2017). Norepinephrine protects against amyloid-induced toxicity via activating the cAMP/PKA signaling pathway by β-adrenergic receptors (Zou et al., 2019). Tooth loss attenuates Fos induction and decreases the pyramidal cell spine number in the hippocampus of SAMP8 mice, resulting in decreased input to the hippocampus (Watanabe et al., 2002; Kubo et al., 2005). Tooth loss in SAMP8 mice also induces a decrease in hippocampal neurons (Onozuka et al., 1999). Therefore, early tooth loss-induced deficits in learning ability and morphologic alterations in the cerebral cortex and hippocampus possibly significantly influenced by the degeneration of neural pathways and/or decrease in input activity. Further studies are required to clarify the causal association between early tooth loss and functional morphologic alterations in the central nervous system related to the pathways innervating the tooth and periodontal ligament.

There is growing consensus that lifetime events including various environmental stressors can increase the risk for developing AD (Wilson et al., 2003; Kang et al., 2007). There is evidence of hypersecretion of glucocorticoids in AD patients (Weiner et al., 1997; Elgh et al., 2006). The psychologic stress could cause cognitive decline (Wilson et al., 2003) and can be associated with an increased risk for AD (Wilson et al., 2007). Environmental stress causes specific alterations in the electrophysiologic properties and neuronal morphology of the brain (McEwen et al., 1999), notably in the hippocampus, which is particularly vulnerable to stress due to the high expression of glucocorticoid receptors. Chronic stress can cause the dendritic atrophy of the hippocampal neurons (Magariños and McEwen, 1995; Sousa et al., 2000), induces morphologic changes of the synaptic terminal structures (Magariños et al., 1997). Restraint stress can induce retraction of the dendritic arbor in the pyramidal neurons of the prefrontal cortex (Perez-Cruz et al., 2009). The cognitive decline induced by stress and/or high glucocorticoid levels are largely concomitantly with a decrease in the hippocampal volume (Landfield et al., 2007; Lupien et al., 2009). The hippocampus shows some neurodegenerative alterations in the early stage of AD. Stress and glucocorticoids also lead to reductions in the volume of the prefrontal cortex (Cerqueira et al., 2007; Schubert et al., 2008), which receives afferent inputs from the hippocampus and is important for modulating the higher cognitive functions. The volume reduction of the hippocampus and prefrontal cortex in depressed patients may be associated with decreased astrocyte density (Rajkowska and Stockmeier, 2013; Cobb et al., 2016), neuronal atrophy (Stockmeier et al., 2004; Rajkowska and Stockmeier, 2013), and declined number and functioning of synapses (Duman and Duman, 2015; Price and Duman, 2020).

Previous reports indicated that chronic stress and exogenous glucocorticoids promote the production and accumulation of Aβ, and induce learning and memory impairment in transgenic AD model mouse (Green et al., 2006; Jeong et al., 2006). The non-transgenic animals treated with chronic stress or exogenous glucocorticoids accelerates intracellular amyloidogenic pathway, promoting Aβ generation (Catania et al., 2009). Animal studies revealed that tau phosphorylation is induced by various stresses like forced swimming in the cold water and food deprivation for a certain period (Härtig et al., 2005; Yoshida et al., 2006). Recent studies reported that emotional stress models demonstrated higher phosphorylated tau levels (Rissman et al., 2007). Restraint is a characteristic emotional stress associated with the pathogenesis of depression (Yoshida et al., 2006). Acute restraint stress causes a reversible elevation in soluble phosphorylated tau, however chronic restraint stress leads to an increase in both soluble and insoluble phosphorylated tau in mouse brain (Rissman et al., 2007).

Chronic stress or glucocorticoid administration increases and activates microglia in the cerebral cortex and hippocampus, and can cause cell death of the hippocampal neurons and astrocytes, in part by inducing neuroinflammation because of the increased concentrations of the pro-inflammatory cytokines in the brain (Al-Ghraiybah et al., 2022). Therefore, stress and glucocorticoids may be closely related to the onset and exacerbation of AD.

Indeed, plasma corticosterone levels were significantly higher in the *App^NL-G-F^*-TE mice than in controls, consistent with our previous findings in SAMP8 mice (Onozuka et al., 2002; Kubo et al., 2014; Katano et al., 2020). Early tooth loss induces an increase in the circulating corticosterone concentrations, hippocampal neuron loss in CA3 region, inhibition of the neuron proliferation in the hippocampal dentate gyrus, and learning impairments in SAMP8 mice (Kubo et al., 2014; Katano et al., 2020). Moreover, the aged mice with early tooth loss have heavier adrenal glands (Onozuka et al., 2000) and elevated blood corticosterone concentrations (Onozuka et al., 2002). These morphologic and physiologic changes induced by tooth loss early in life are similar to the alterations caused by chronic stress (Luine et al., 1994). The morphologic changes observed in the *App^NL-G-F^*-TE mice in the present study closely resemble the stress-induced morphologic changes in the cerebral cortex and hippocampus of AD (Härtig et al., 2005; Green et al., 2006; Jeong et al., 2006; Yoshida et al., 2006; Rissman et al., 2007; Catania et al., 2009). Therefore, early life tooth loss in the *App^NL-G-F^* mice presumably acts as a chronic stressor, which exacerbates AD. Further studies are needed to determine the precise causal relationship between early tooth loss and the onset and exacerbation of AD.

In summary, our findings provide evidence that long-term tooth loss represents a chronic stressor, activating the recruitment of microglia and astrocytes, increasing the secretion of pro IL-1β and TNFα; exacerbating neuroinflammation, Aβ deposition, phosphorylated tau protein; and leading to synaptic dysfunction and spatial learning impairments in AD model mice.

## Data availability statement

The original contributions presented in the study are included in the article/Supplementary material, further inquiries can be directed to the corresponding author.

## Ethics statement

The animal study was approved by the ethics committee for animal care and experimentation at Asahi University School of Dentistry (permission code: 20–024). The study was conducted in accordance with the local legislation and institutional requirements.

## Author contributions

SO: Conceptualization, Investigation, Visualization, Writing – original draft, Writing – review & editing. KY: Investigation, Writing – review & editing. TS: Writing – review & editing. TCS: Writing – review & editing. MI: Writing – review & editing. KA: Conceptualization, Investigation, Validation, Writing – original draft, Writing – review & editing. K-YK: Conceptualization, Investigation, Supervision, Writing – original draft, Writing – review & editing.
