# Supplementary material for: Effects of early tooth loss on chronic stress and progression of neuropathogenesis of Alzheimer’s disease in adult Alzheimer’s model AppNL-G-F mice
Source: Front Aging Neurosci. 2024 Feb 26;16:1361847. doi: 10.3389/fnagi.2024.1361847 (PMC10925668; doi:10.3389/fnagi.2024.1361847)
Supplement: Supplementary file 3 [file Presentation_1.PPTX]

## Slide 1
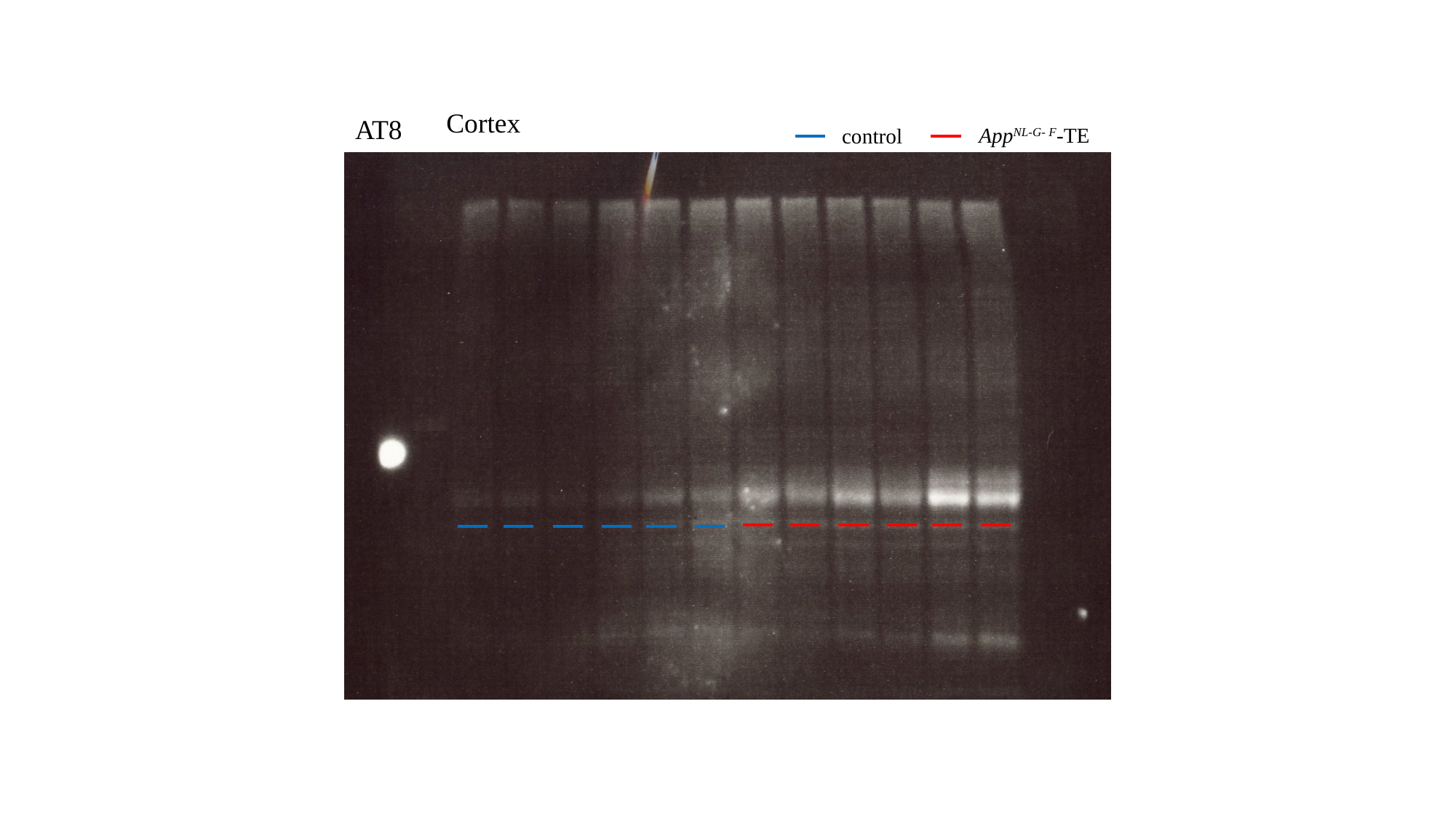

Cortex
AT8
AppNL-G- F-TE
control

## Slide 2
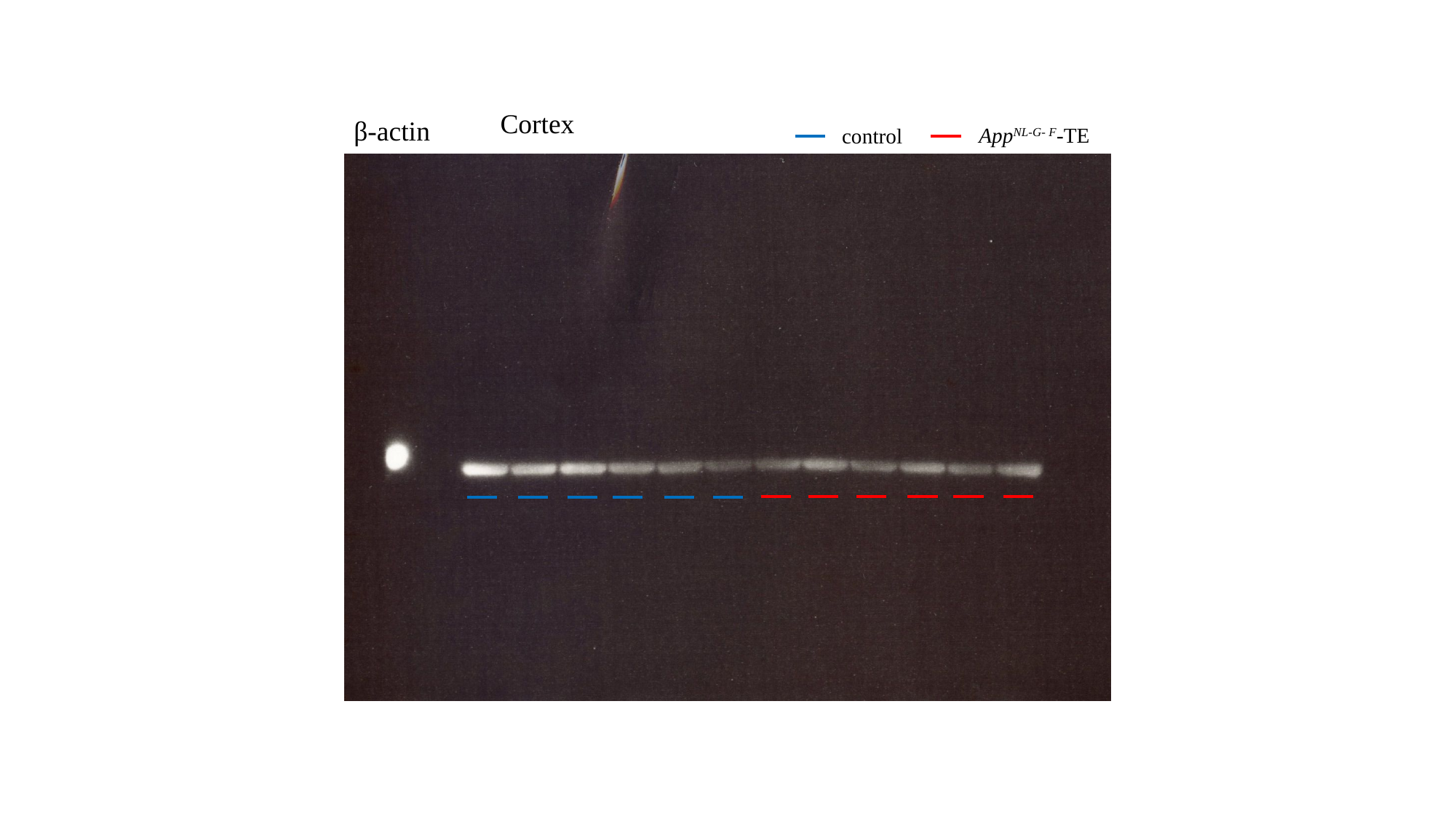

Cortex
β-actin
AppNL-G- F-TE
control

## Slide 3
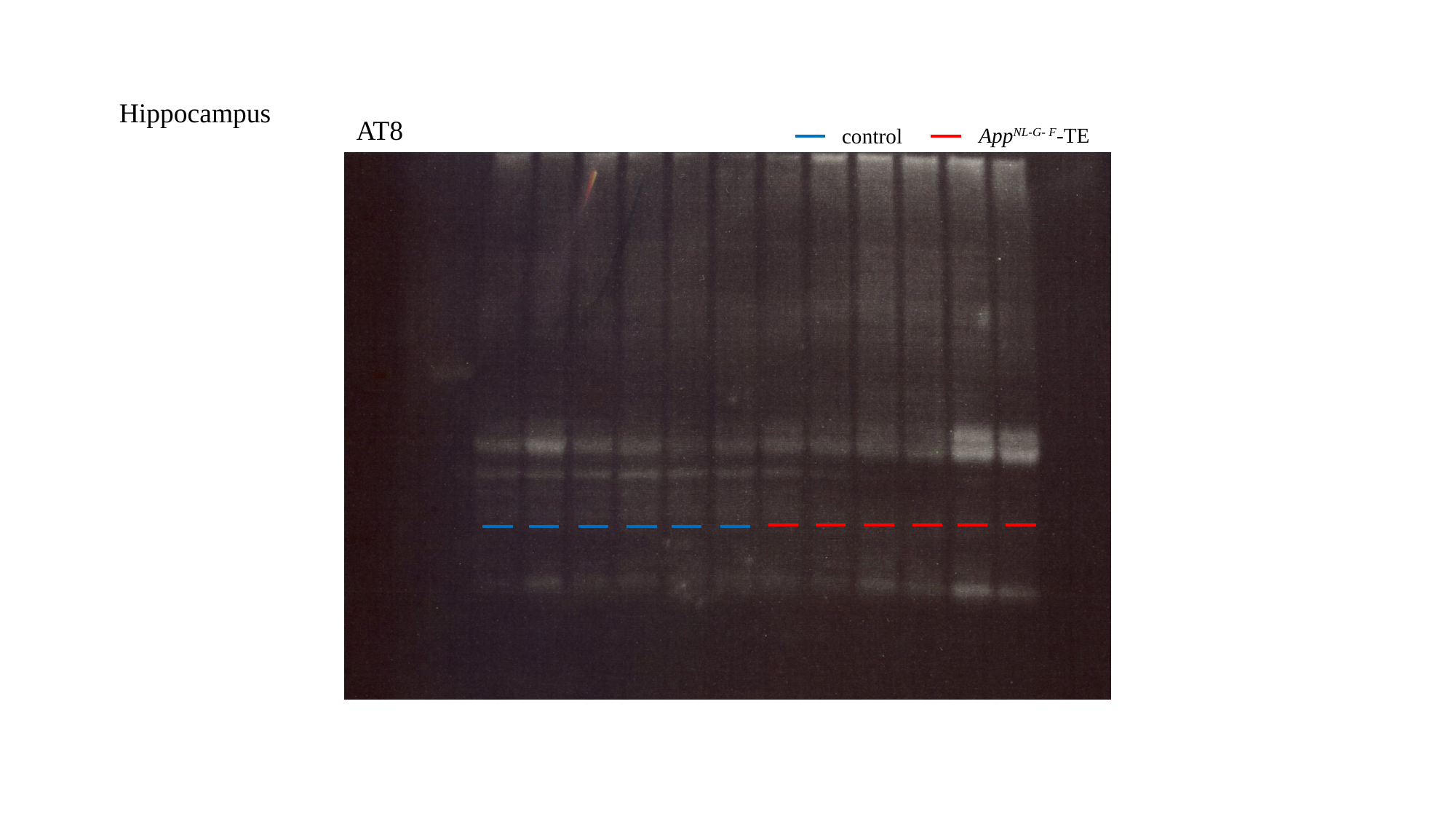

Hippocampus
AT8
AppNL-G- F-TE
control

## Slide 4
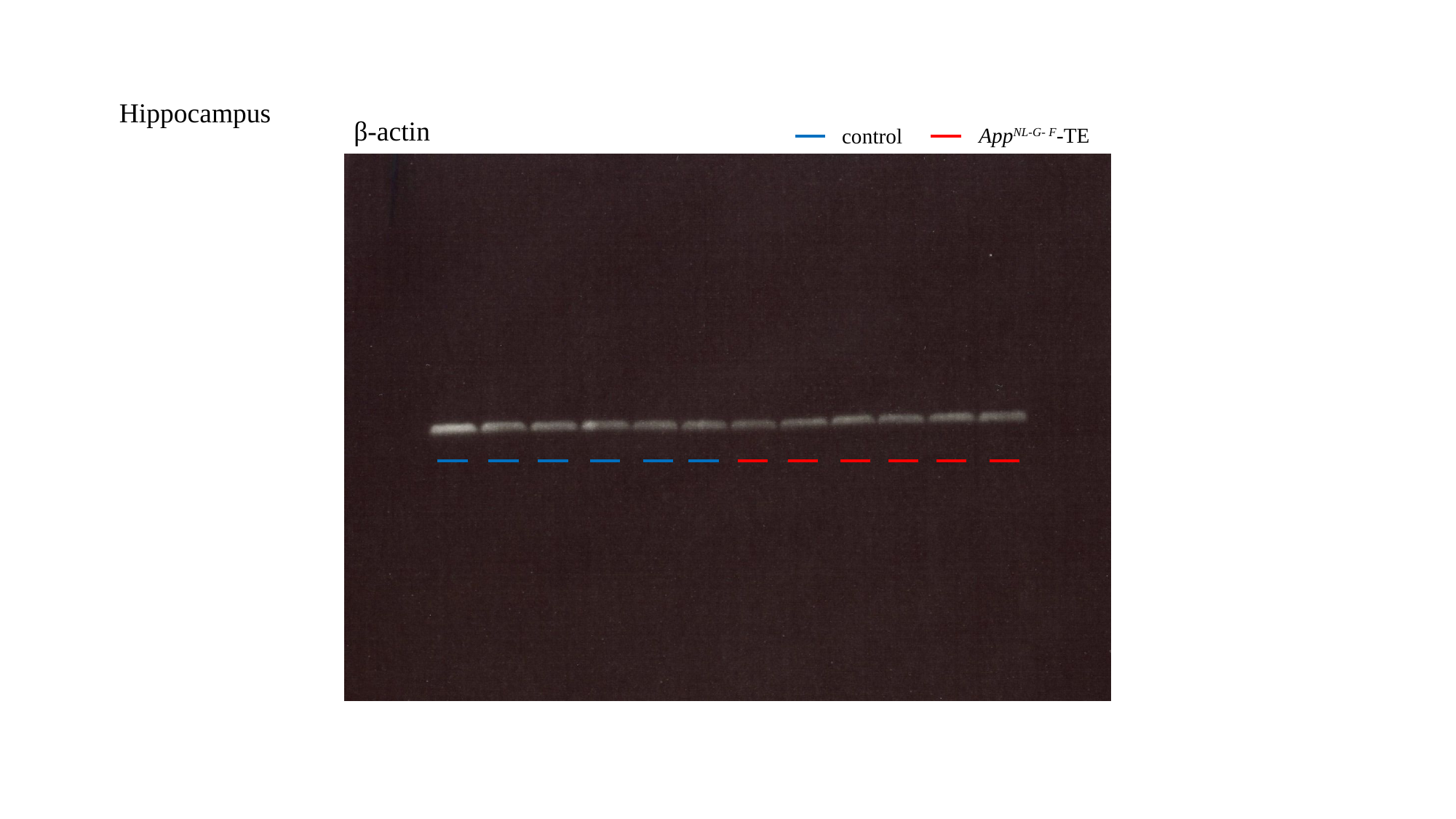

Hippocampus
β-actin
AppNL-G- F-TE
control

## Slide 5
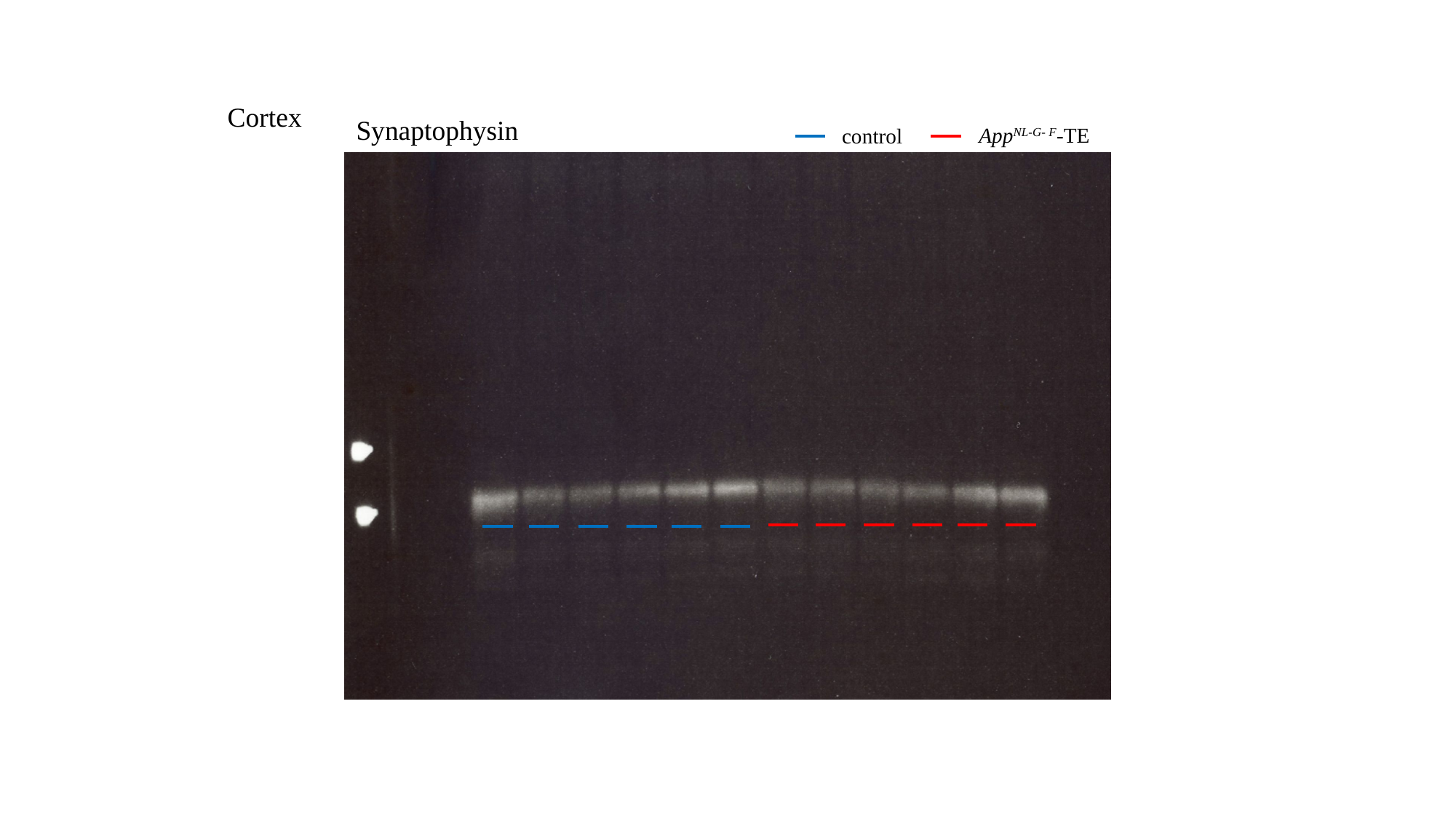

Cortex
Synaptophysin
AppNL-G- F-TE
control

## Slide 6
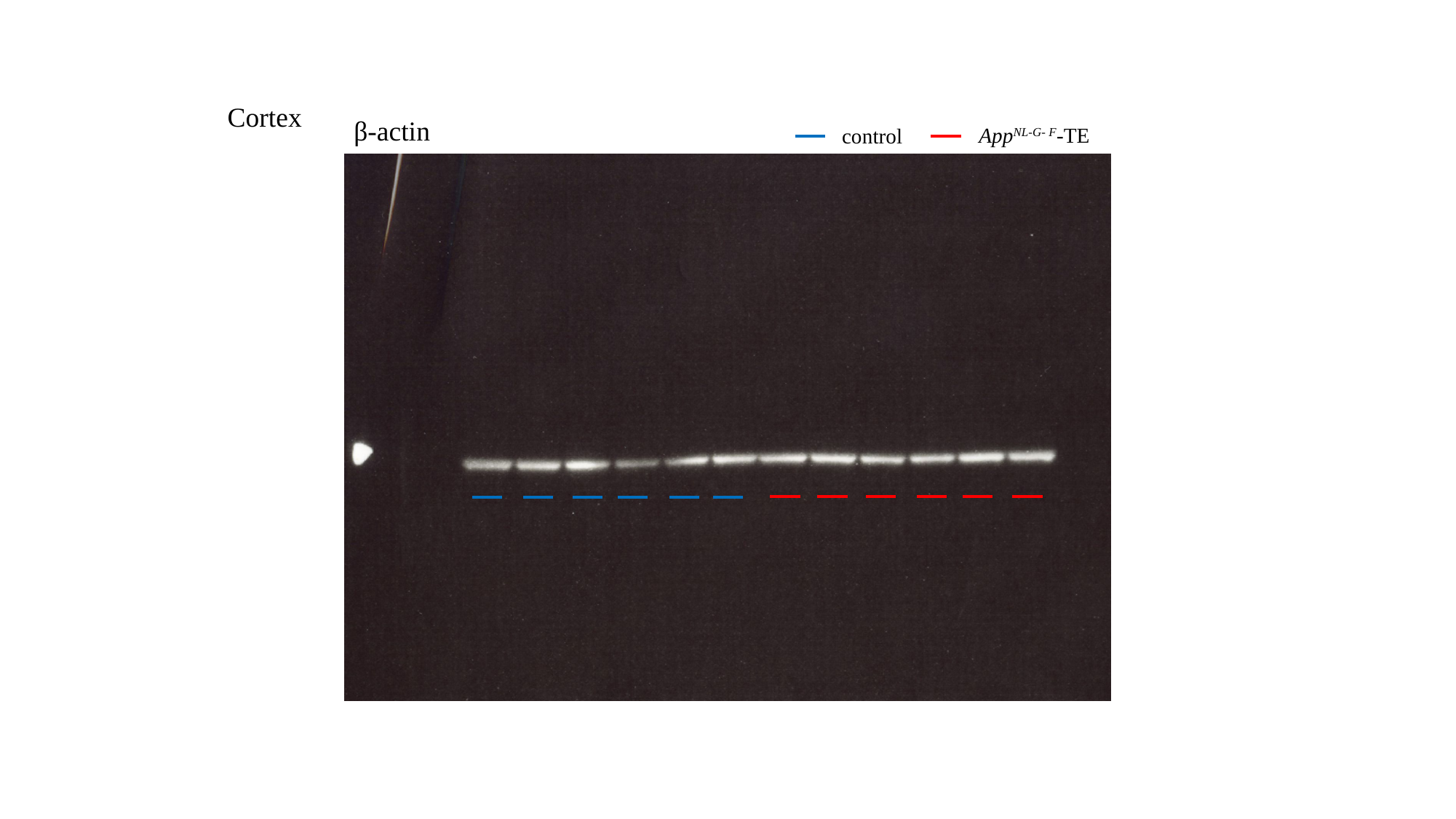

Cortex
β-actin
AppNL-G- F-TE
control

## Slide 7
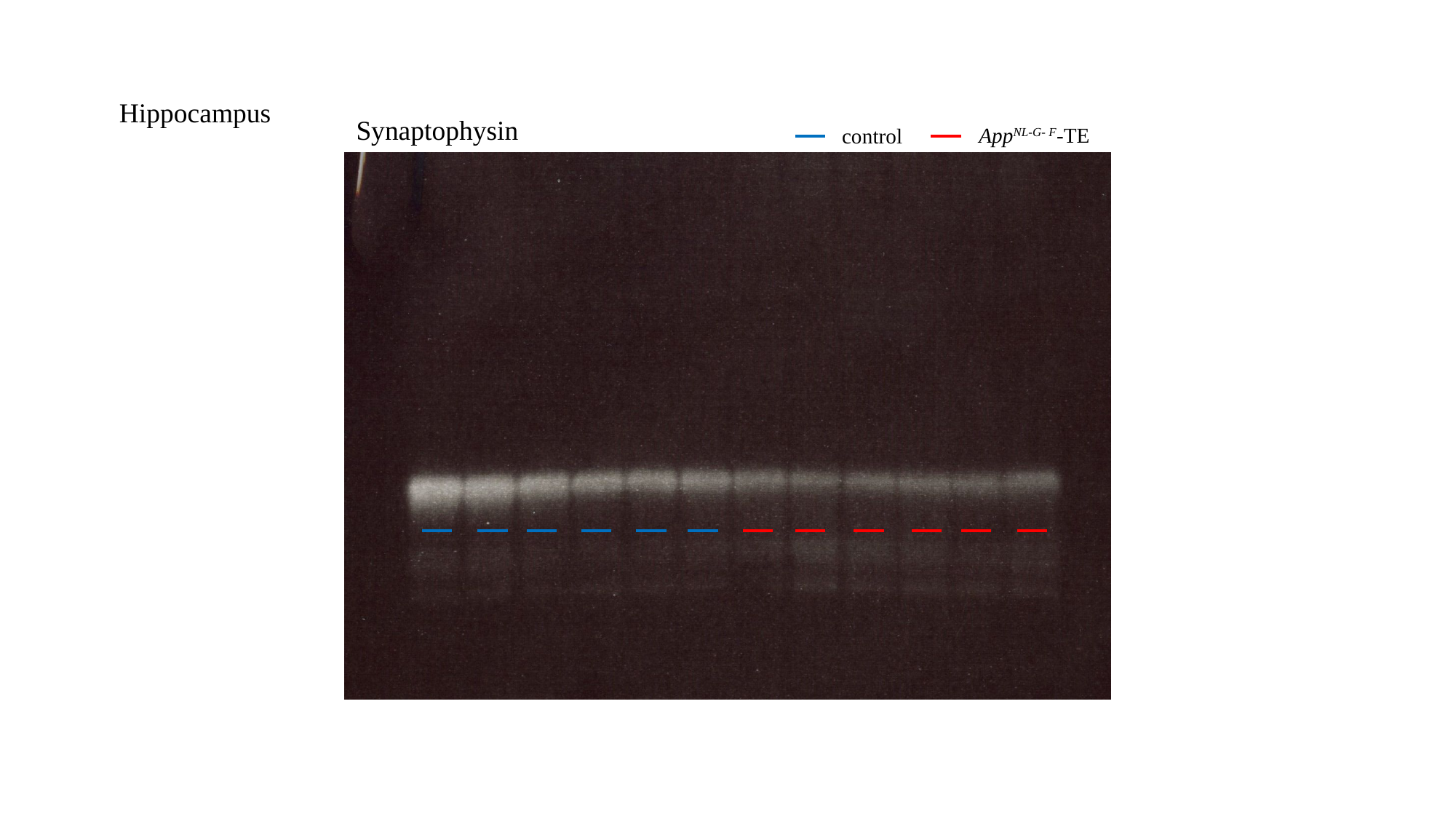

Hippocampus
Synaptophysin
AppNL-G- F-TE
control

## Slide 8
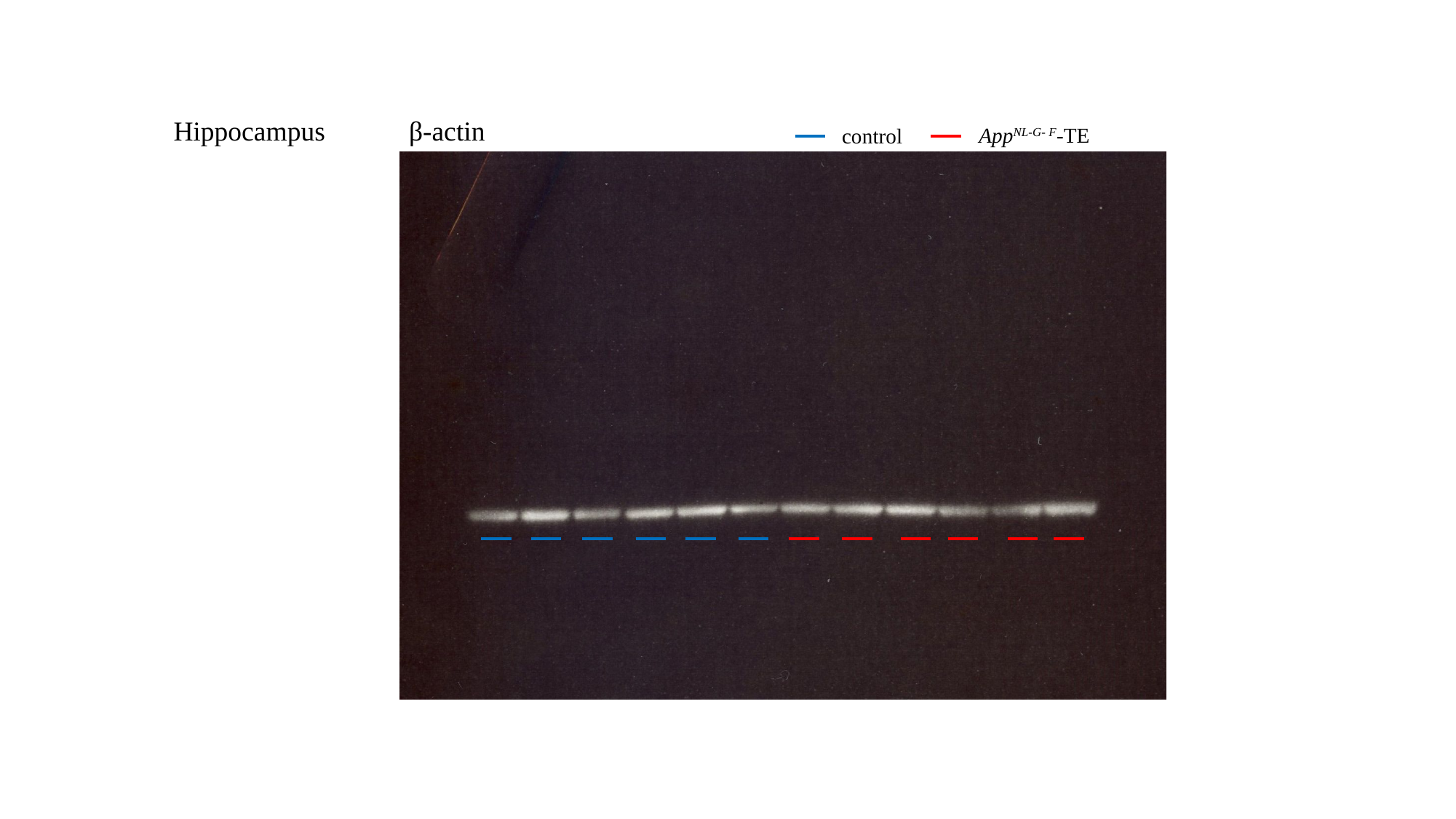

Hippocampus
β-actin
AppNL-G- F-TE
control
