# Supplementary material for: Effects of early tooth loss on chronic stress and progression of neuropathogenesis of Alzheimer’s disease in adult Alzheimer’s model AppNL-G-F mice
Source: Front Aging Neurosci. 2024 Feb 26;16:1361847. doi: 10.3389/fnagi.2024.1361847 (PMC10925668; doi:10.3389/fnagi.2024.1361847)
Supplement: Supplementary file 4 [file Presentation_2.PPTX]

## Slide 1
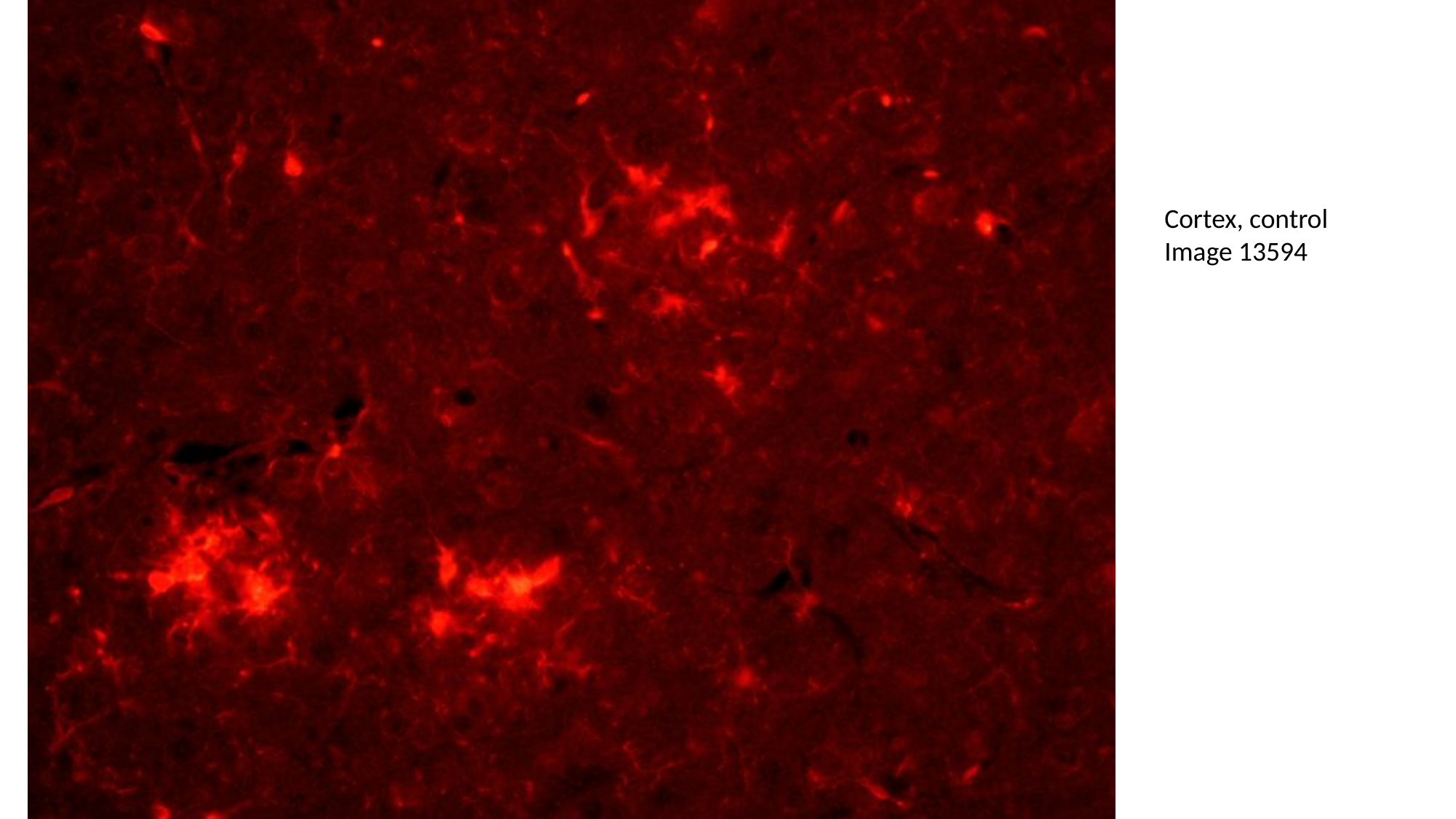

Cortex, control
Image 13594

## Slide 2
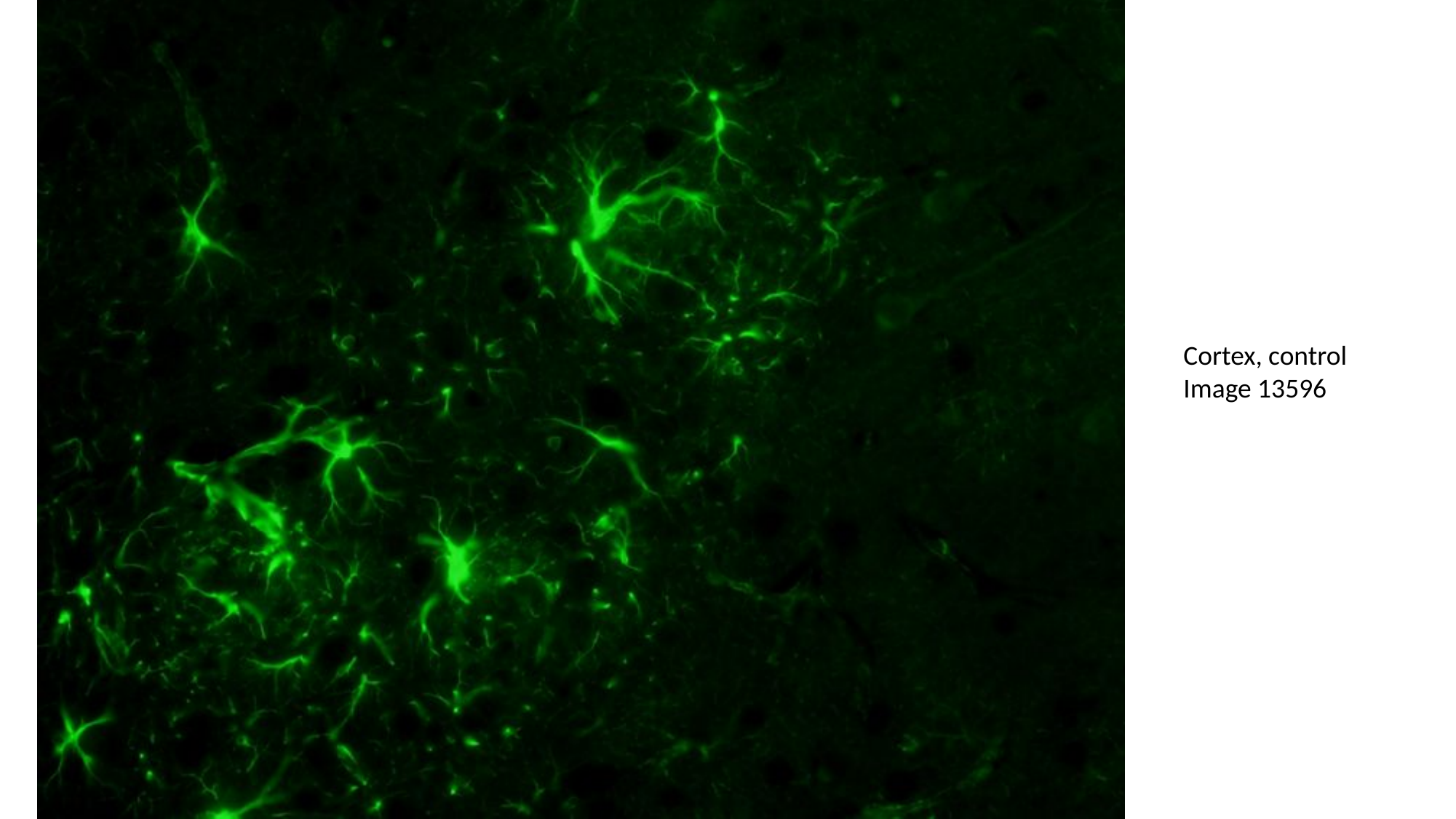

Cortex, control
Image 13596

## Slide 3
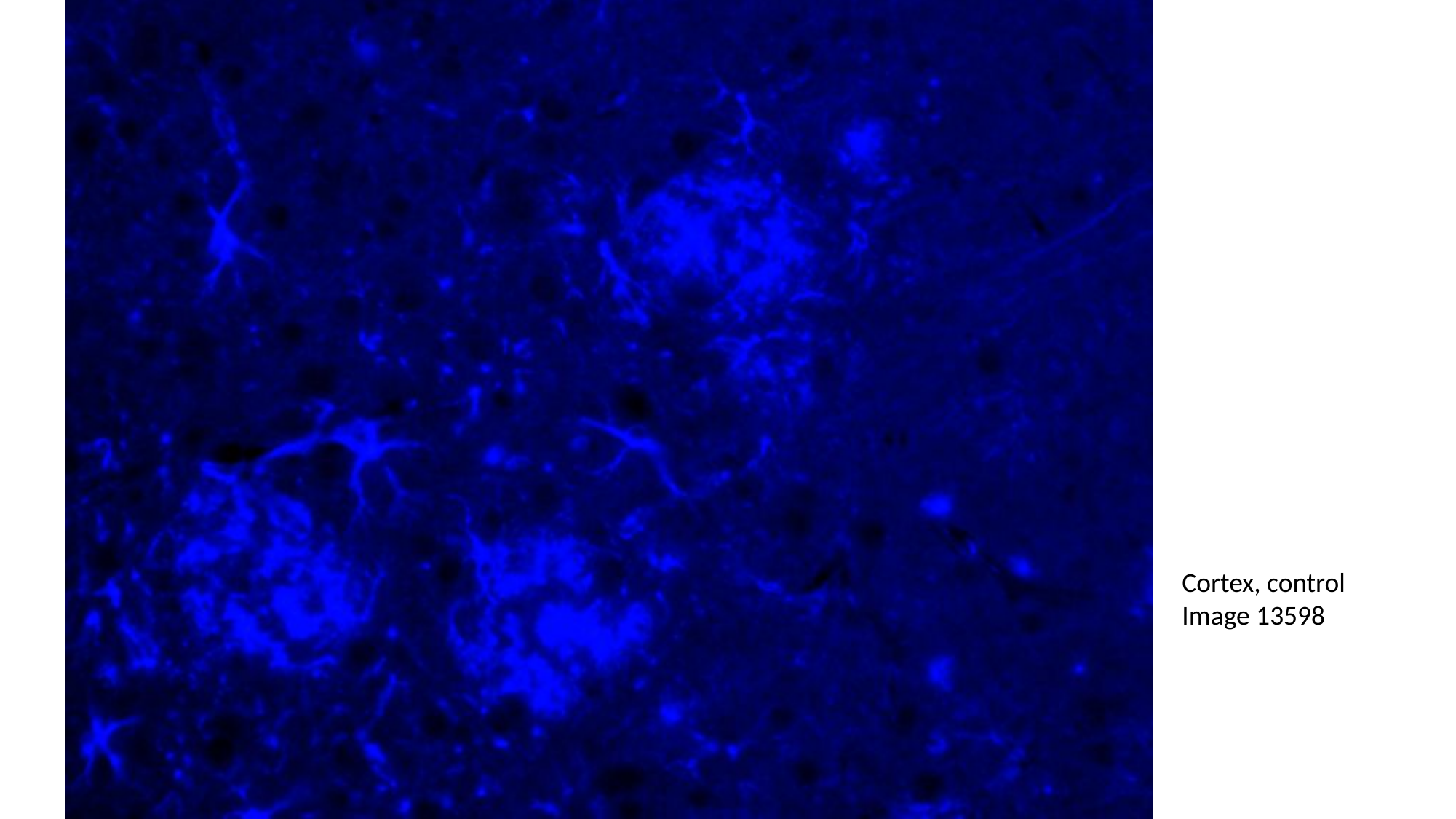

Cortex, control
Image 13598

## Slide 4
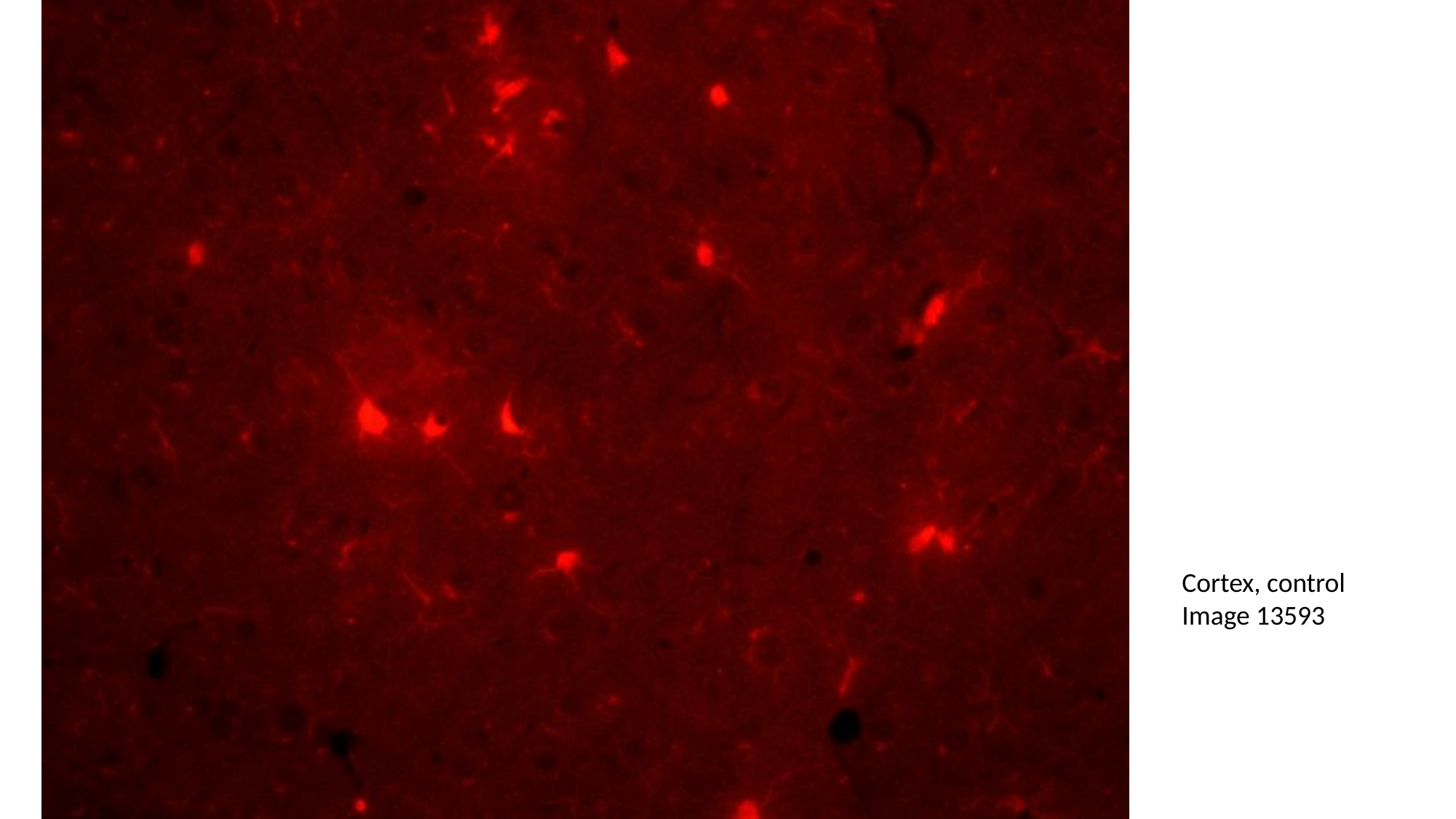

Cortex, control
Image 13593

## Slide 5
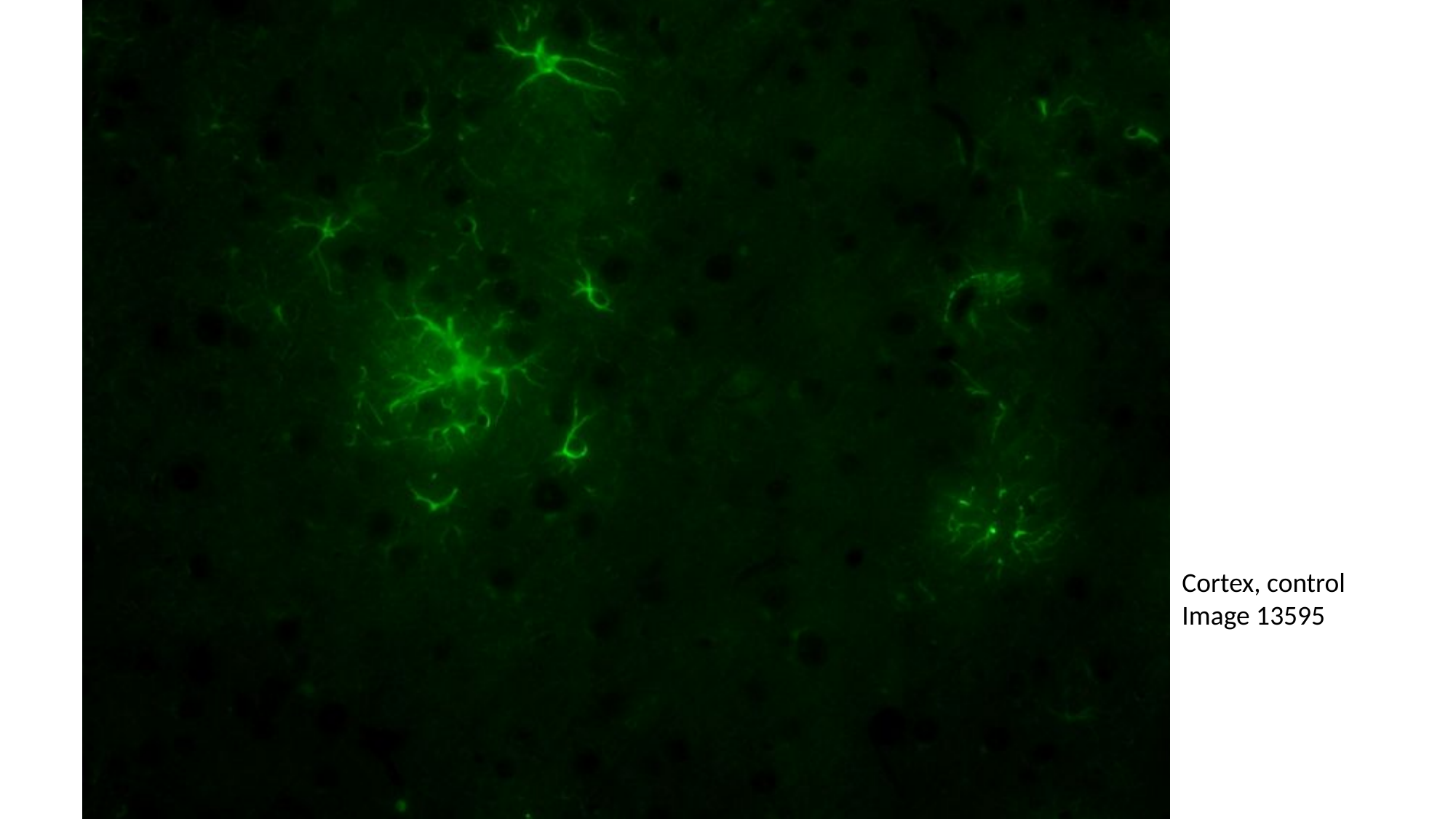

Cortex, control
Image 13595

## Slide 6
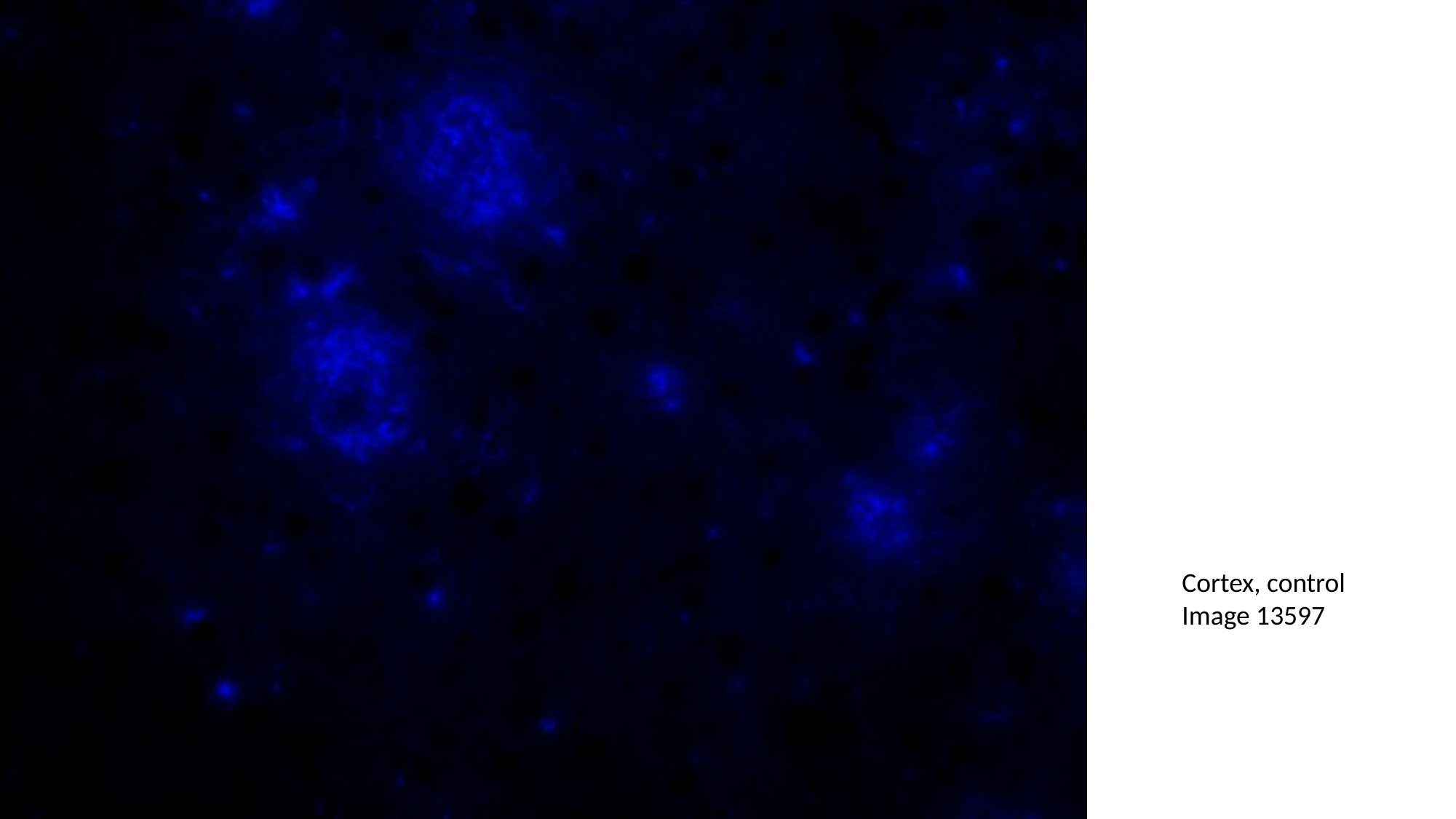

Cortex, control
Image 13597

## Slide 7
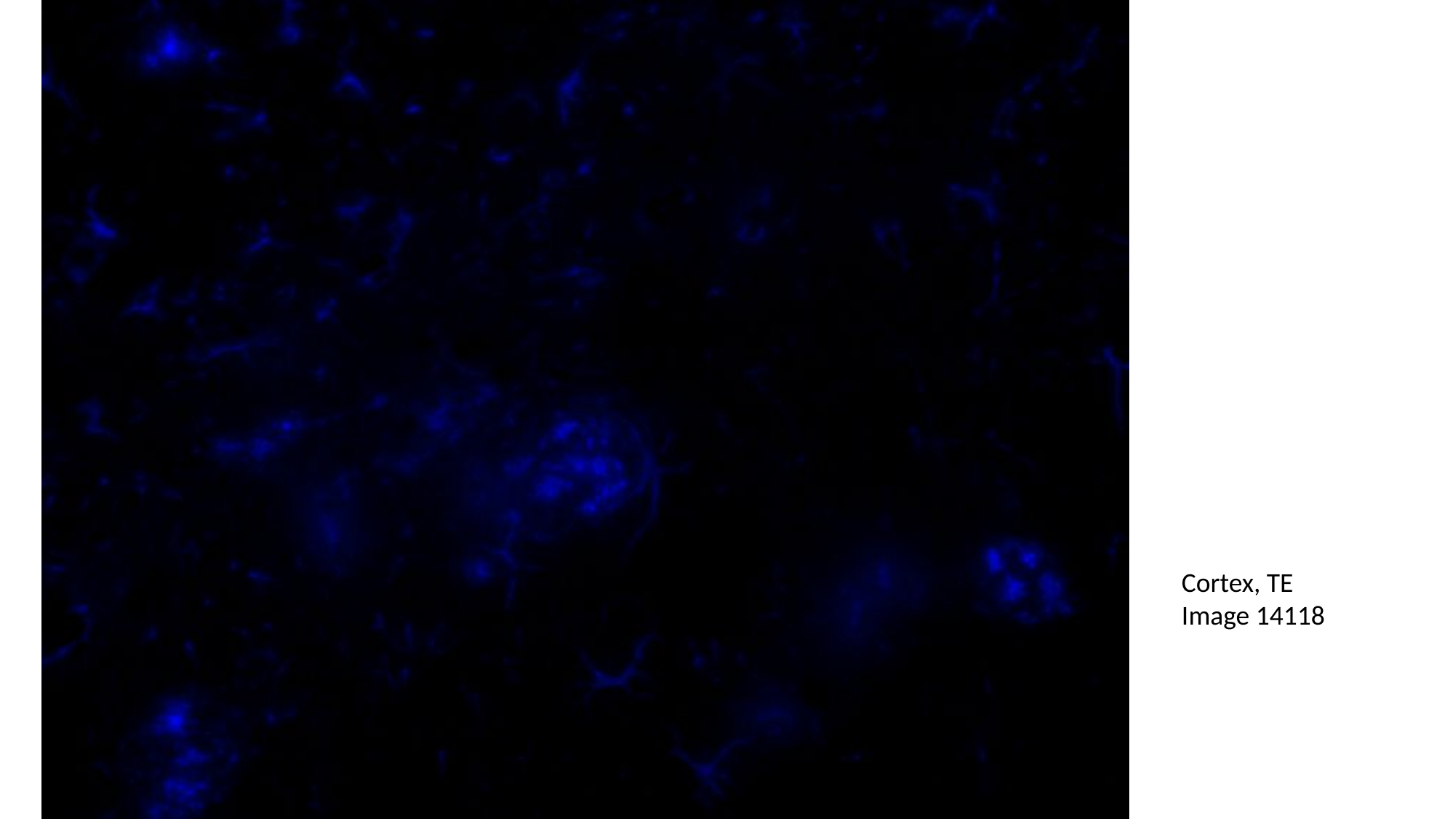

Cortex, TE
Image 14118

## Slide 8
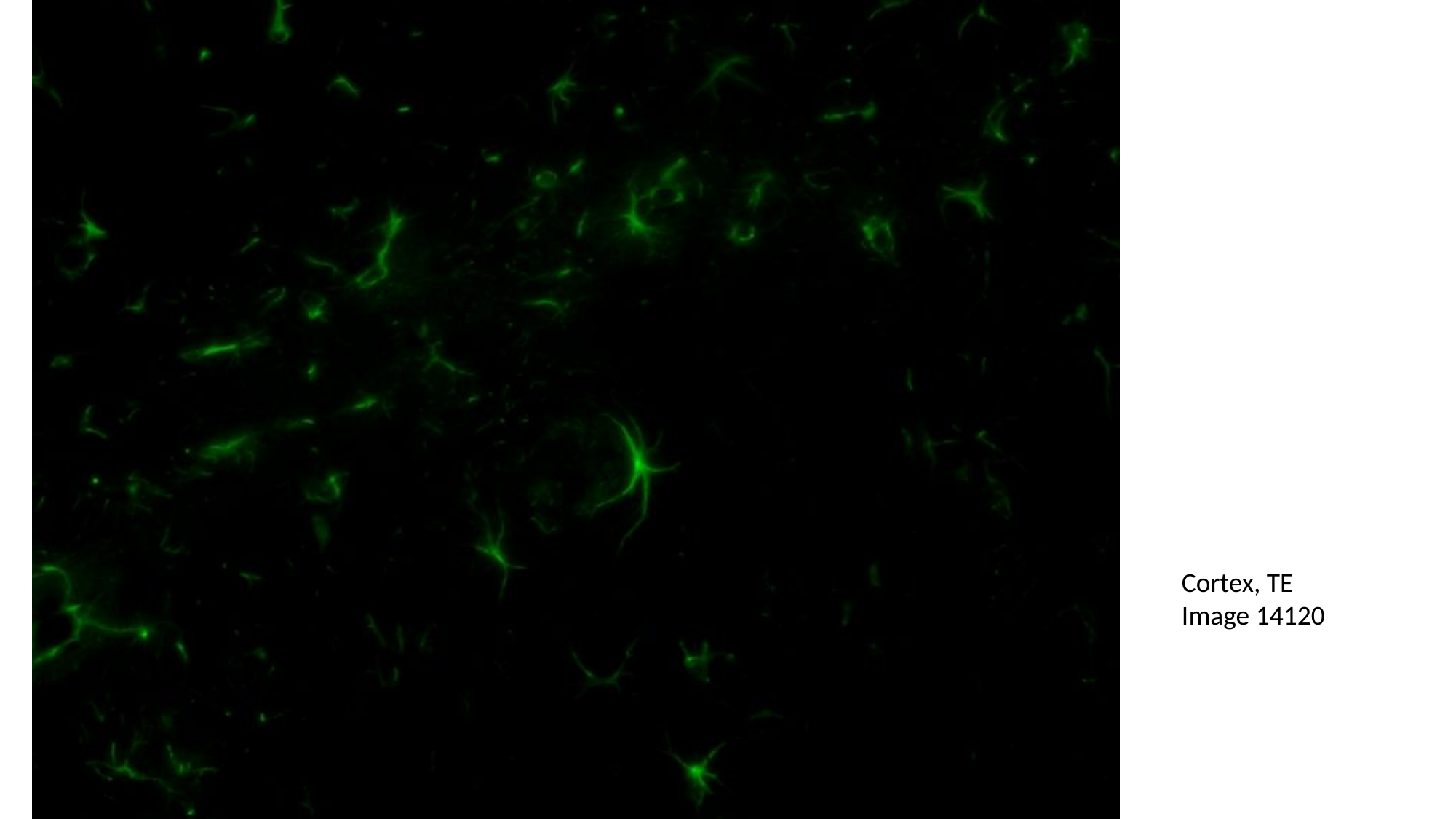

Cortex, TE
Image 14120

## Slide 9
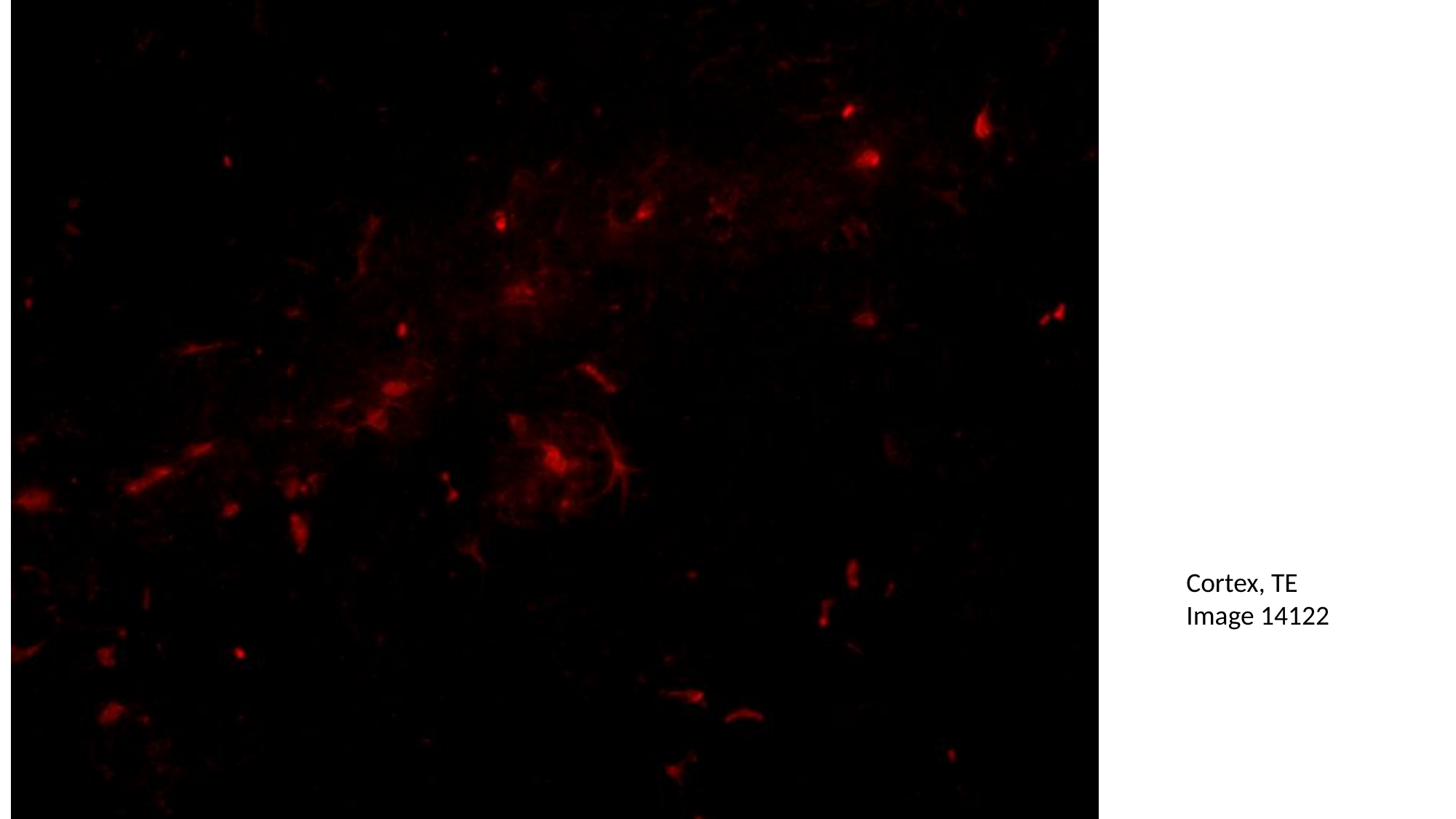

Cortex, TE
Image 14122

## Slide 10
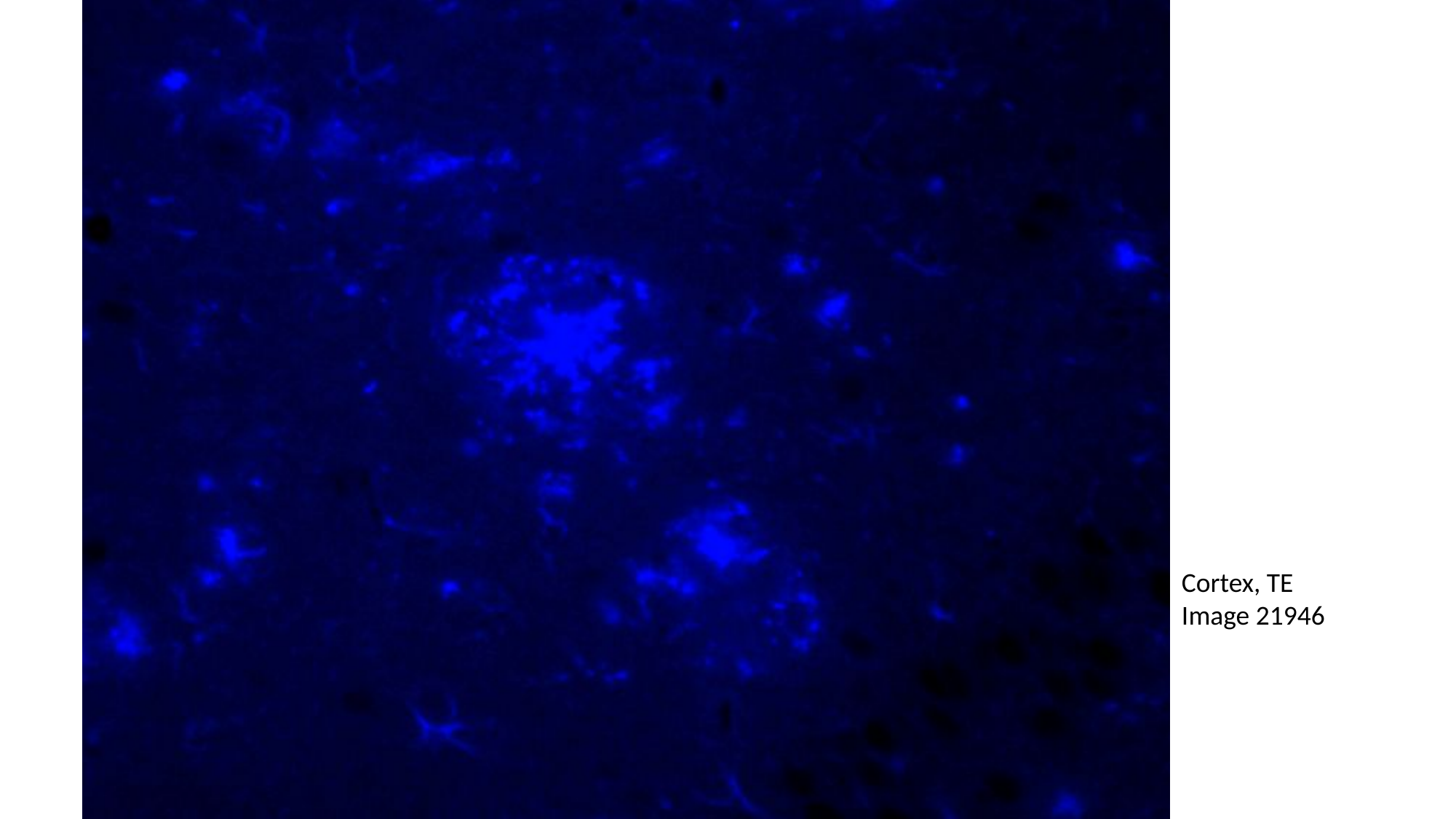

Cortex, TE
Image 21946

## Slide 11
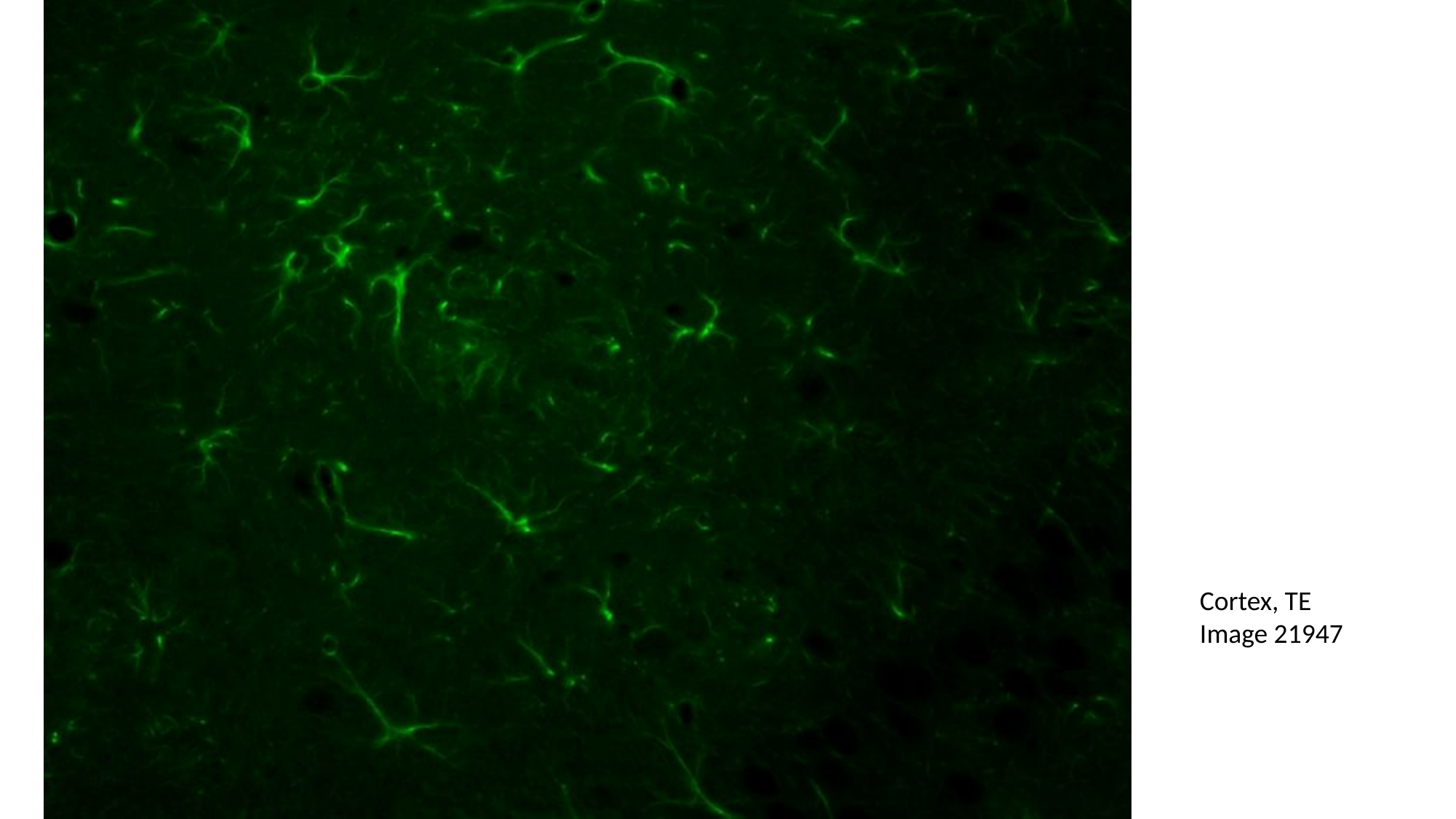

Cortex, TE
Image 21947

## Slide 12
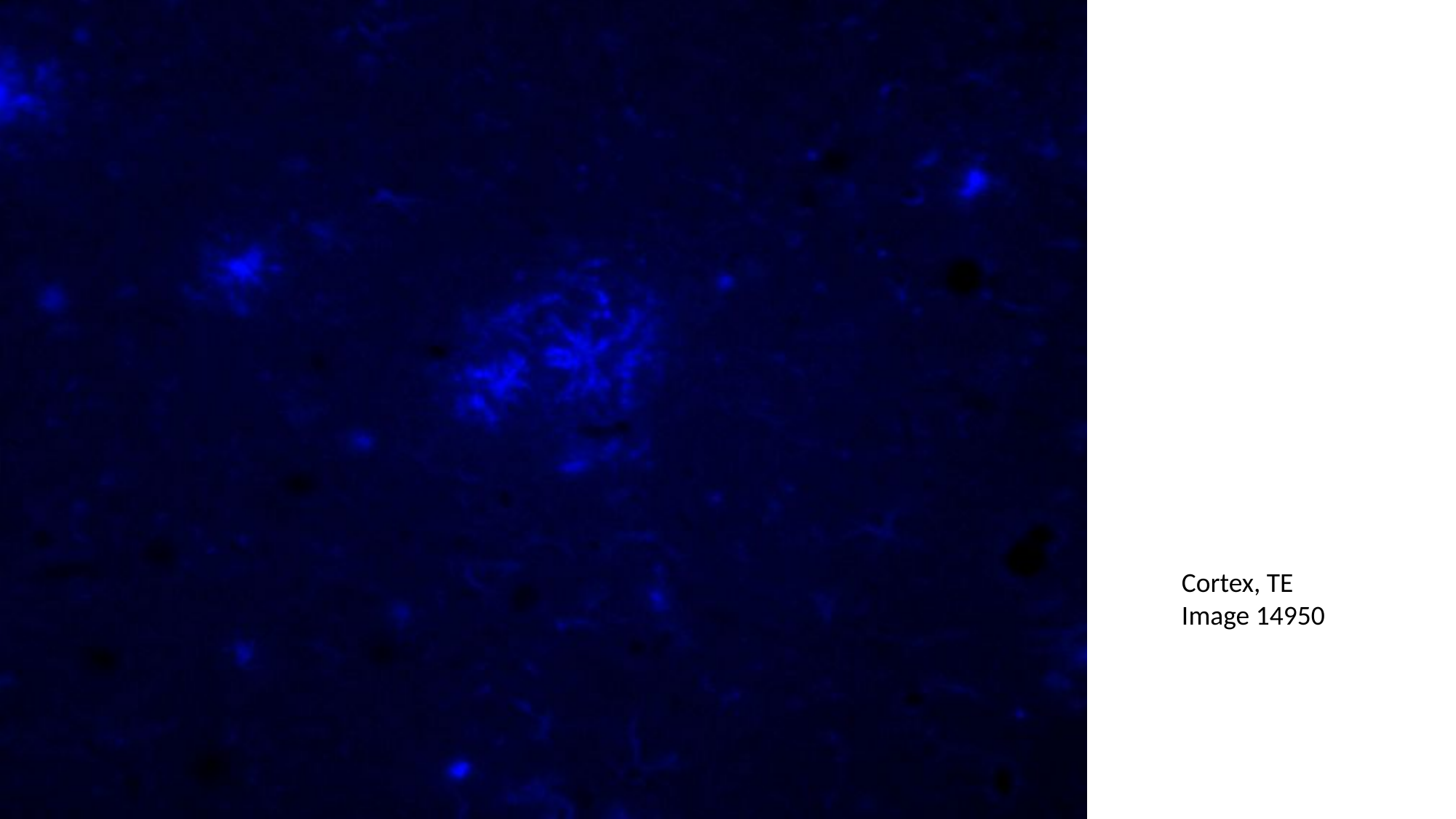

Cortex, TE
Image 14950
