# Supplementary material for: Effects of early tooth loss on chronic stress and progression of neuropathogenesis of Alzheimer’s disease in adult Alzheimer’s model AppNL-G-F mice
Source: Front Aging Neurosci. 2024 Feb 26;16:1361847. doi: 10.3389/fnagi.2024.1361847 (PMC10925668; doi:10.3389/fnagi.2024.1361847)
Supplement: Supplementary file 5 [file Presentation_3.PPTX]

## Slide 1
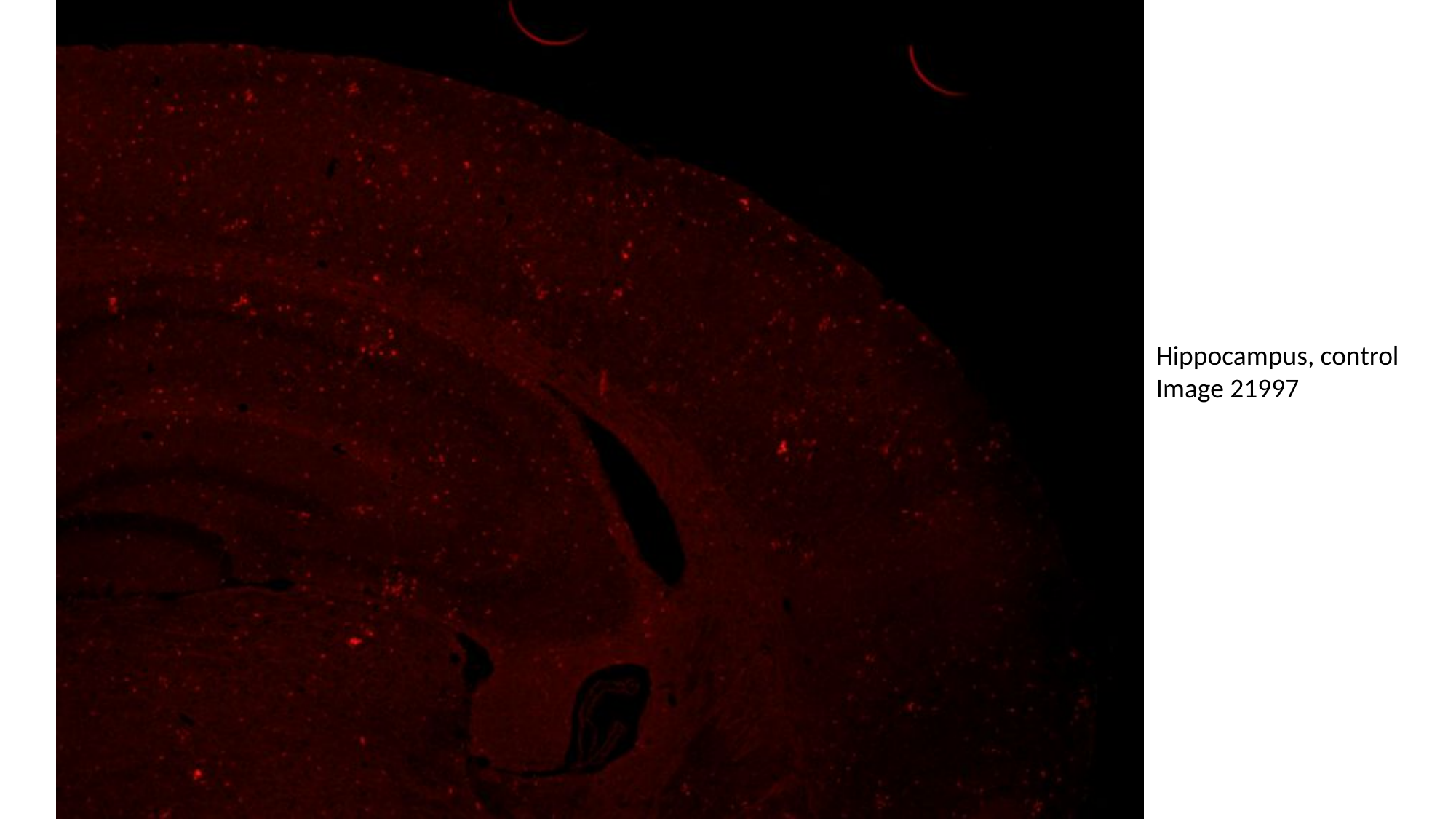

Hippocampus, control
Image 21997

## Slide 2
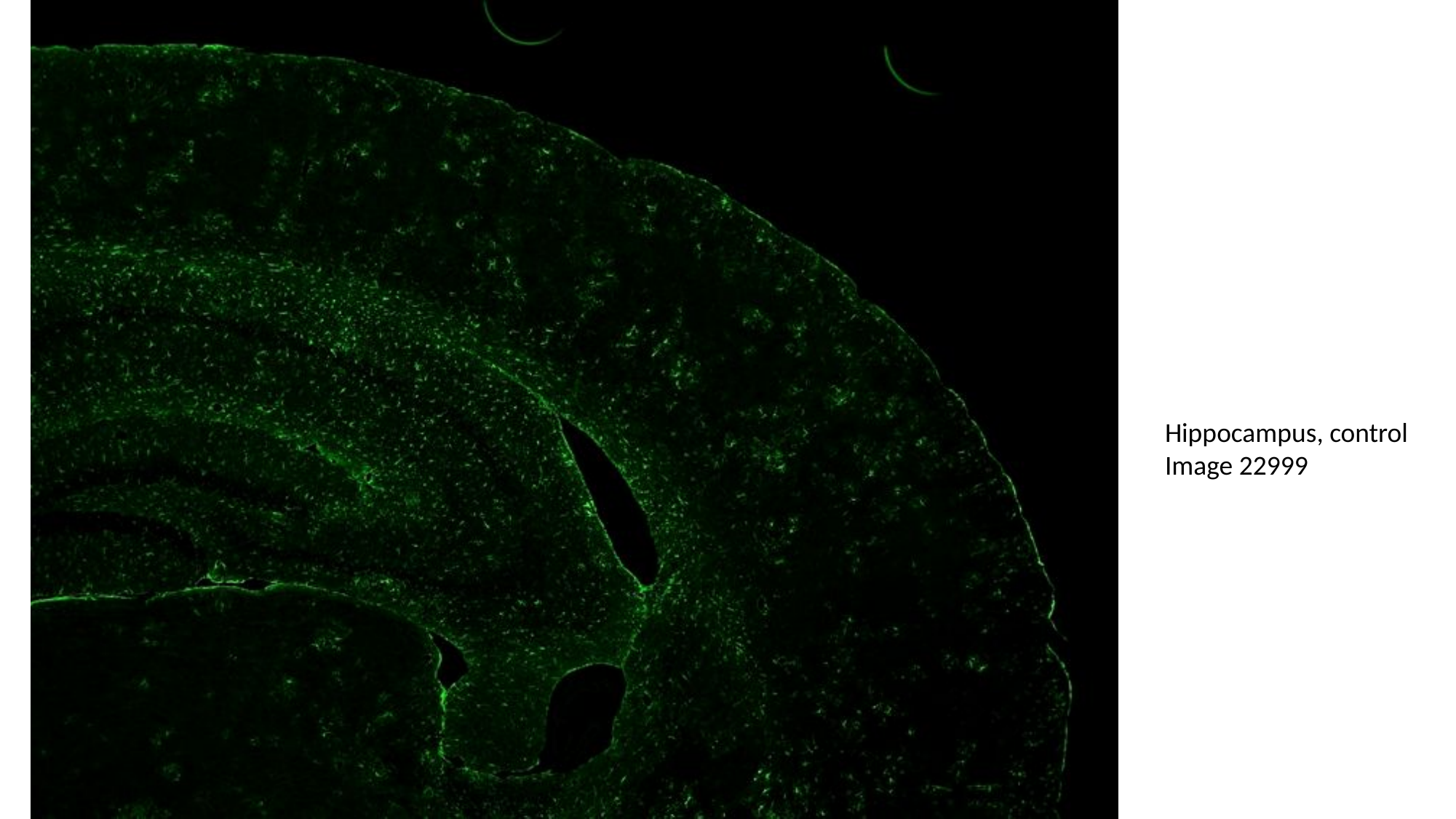

Hippocampus, control
Image 22999

## Slide 3
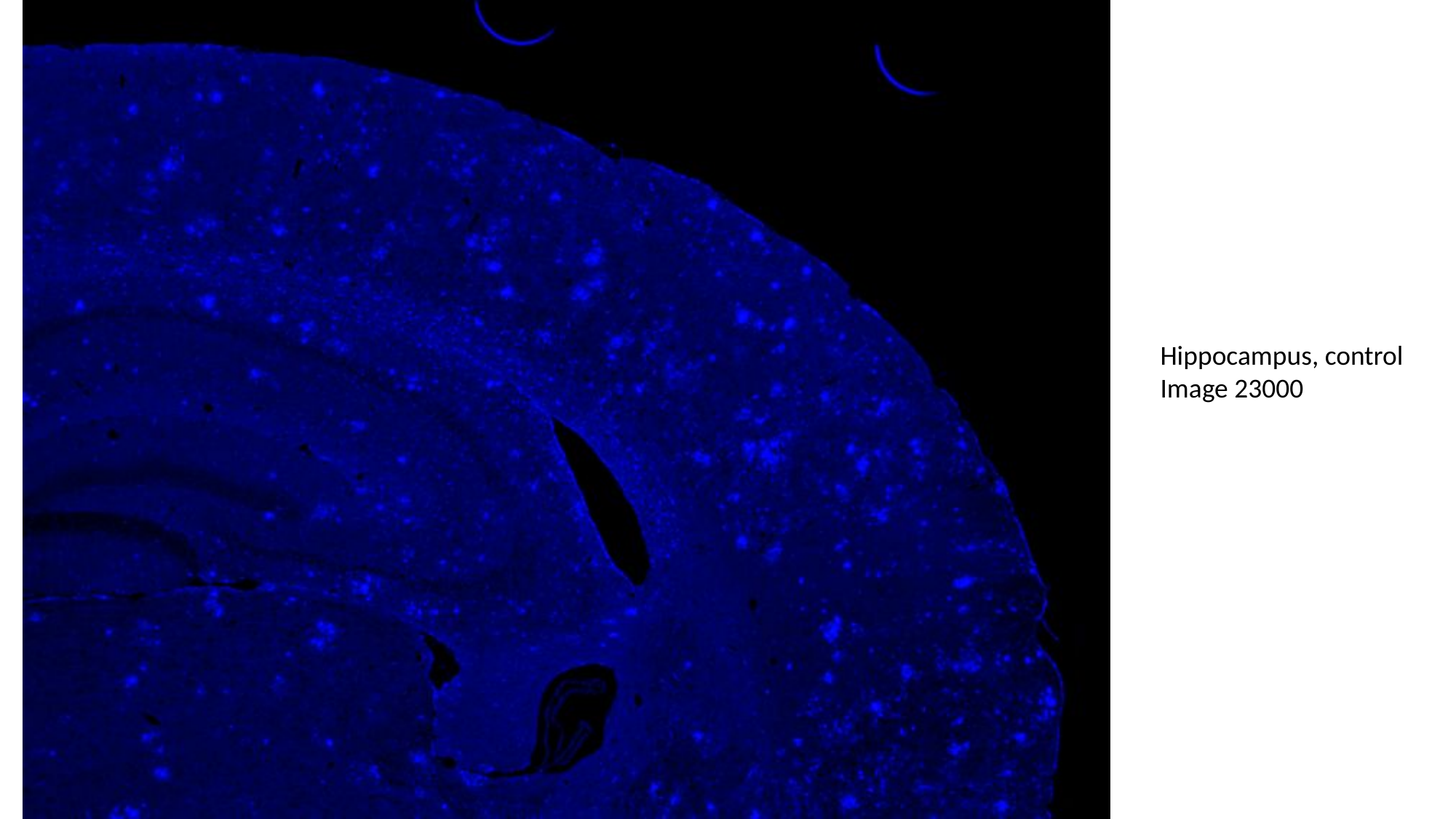

Hippocampus, control
Image 23000

## Slide 4
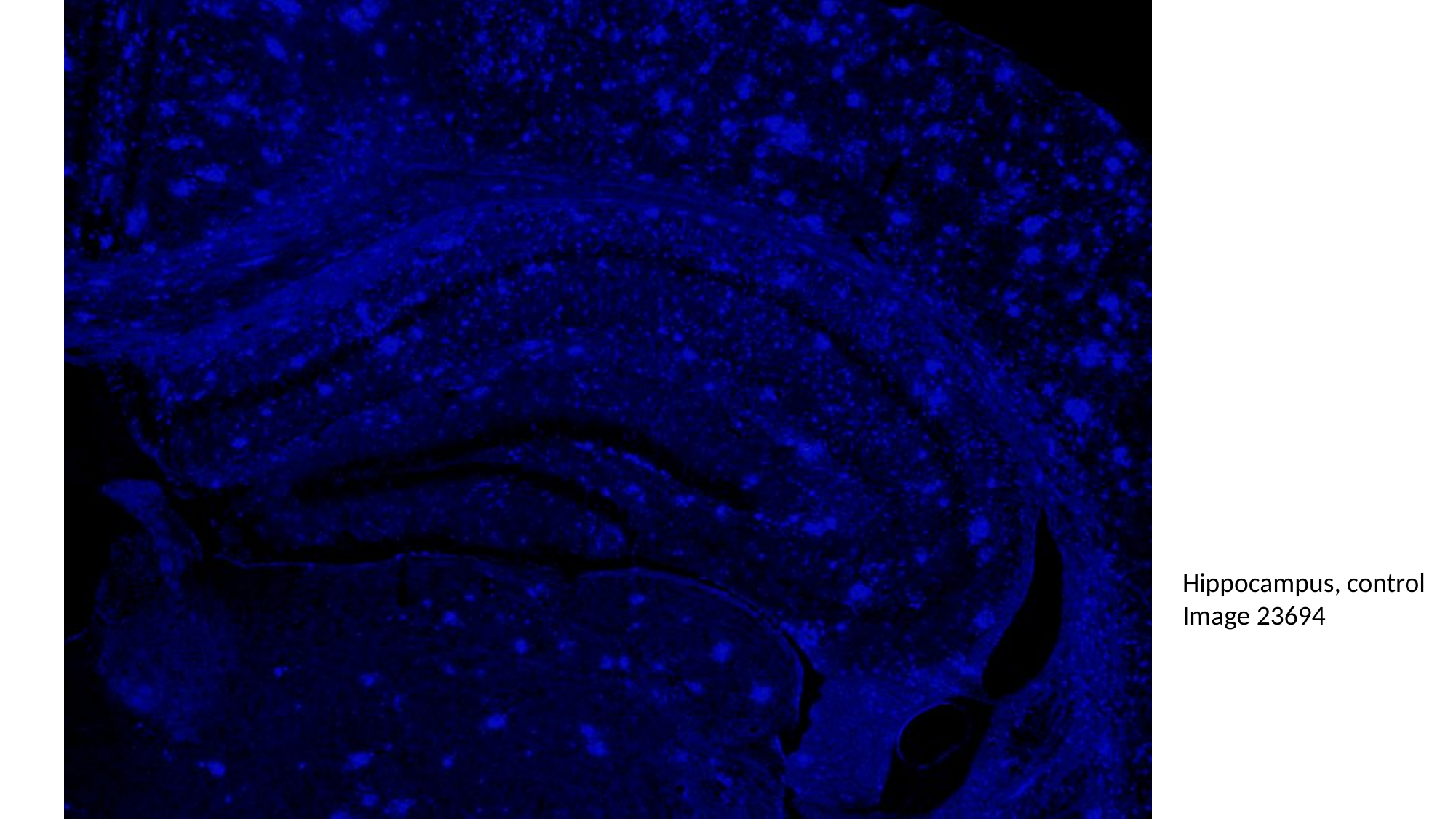

Hippocampus, control
Image 23694

## Slide 5
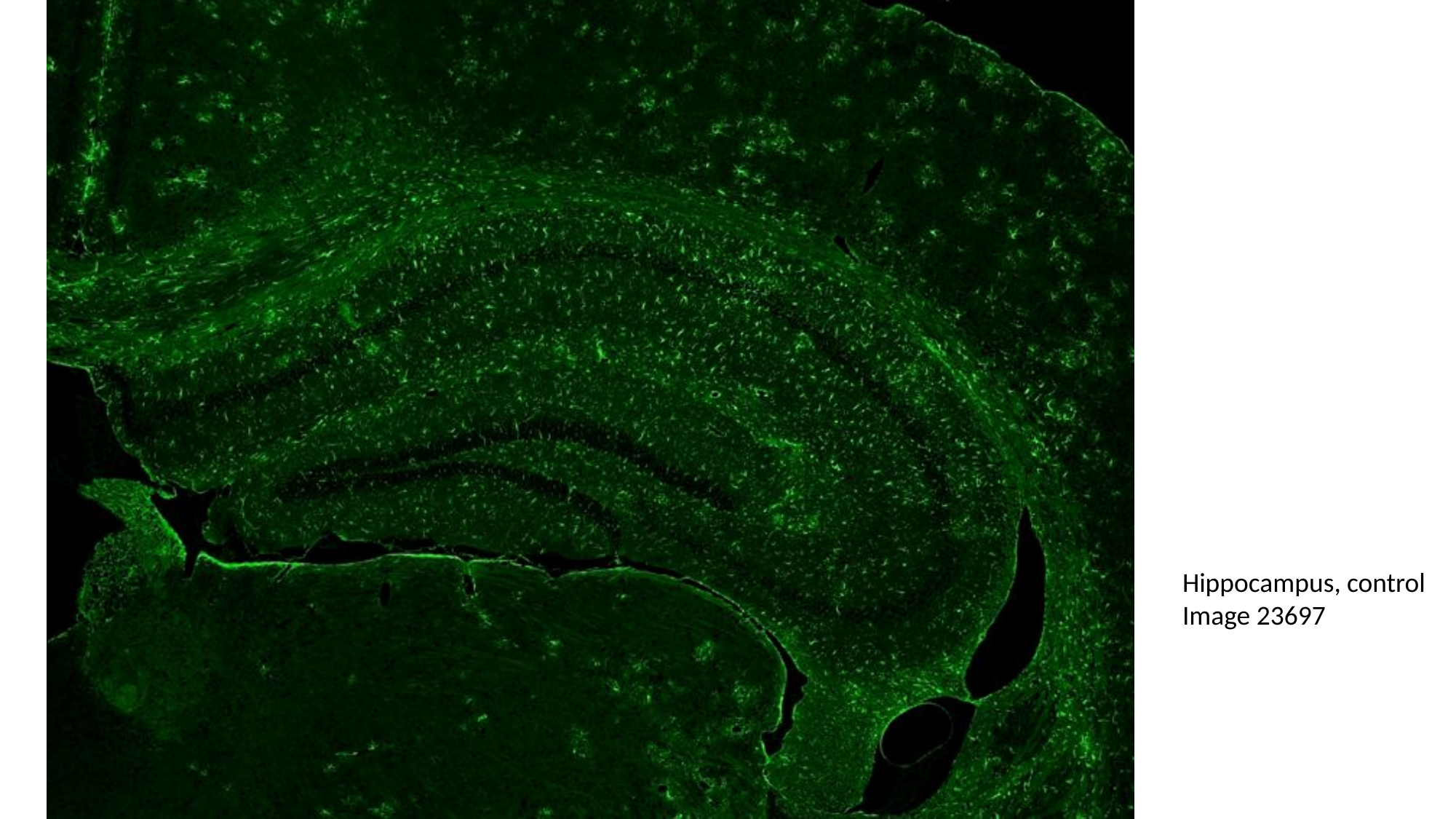

Hippocampus, control
Image 23697

## Slide 6
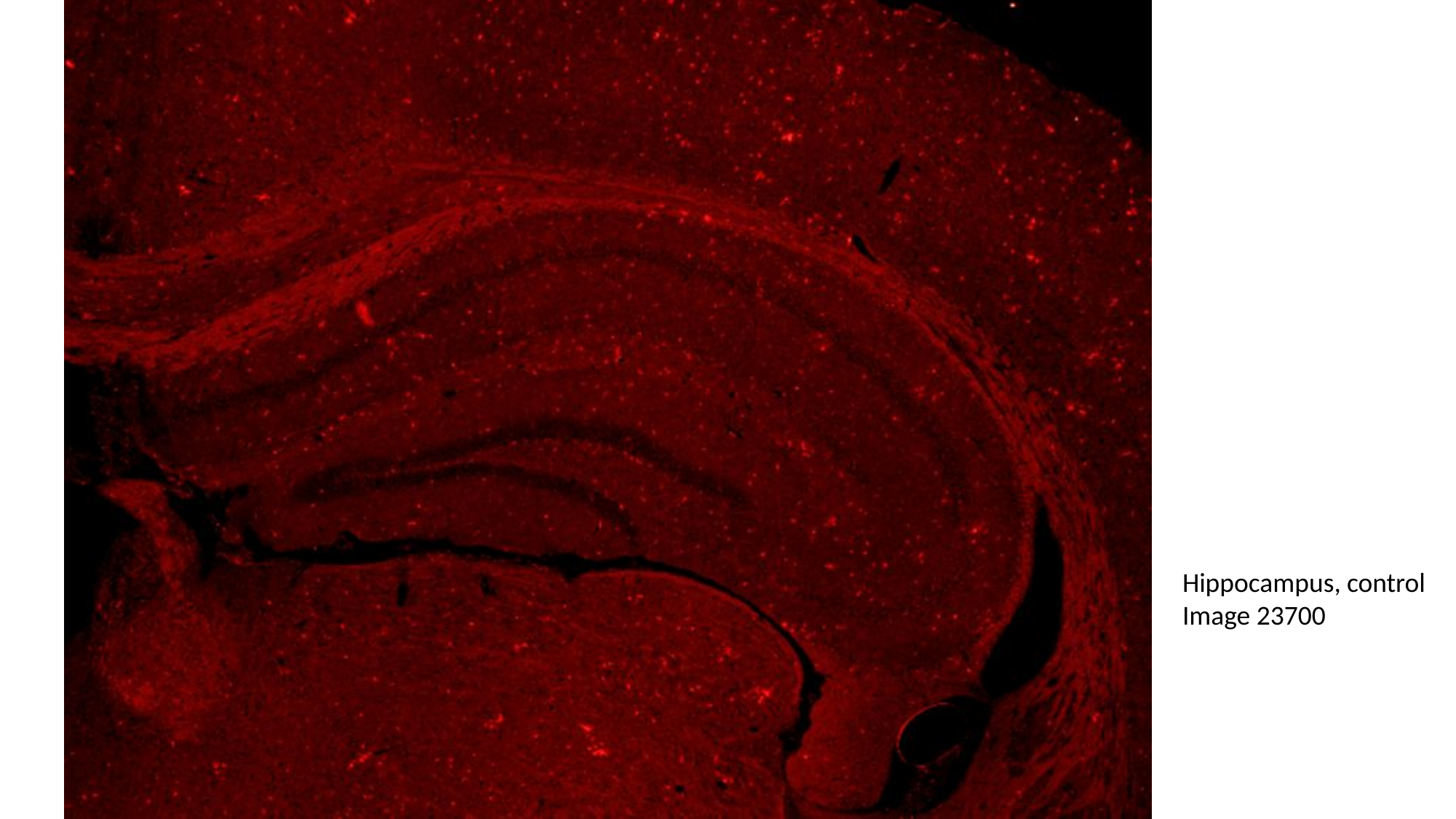

Hippocampus, control
Image 23700

## Slide 7
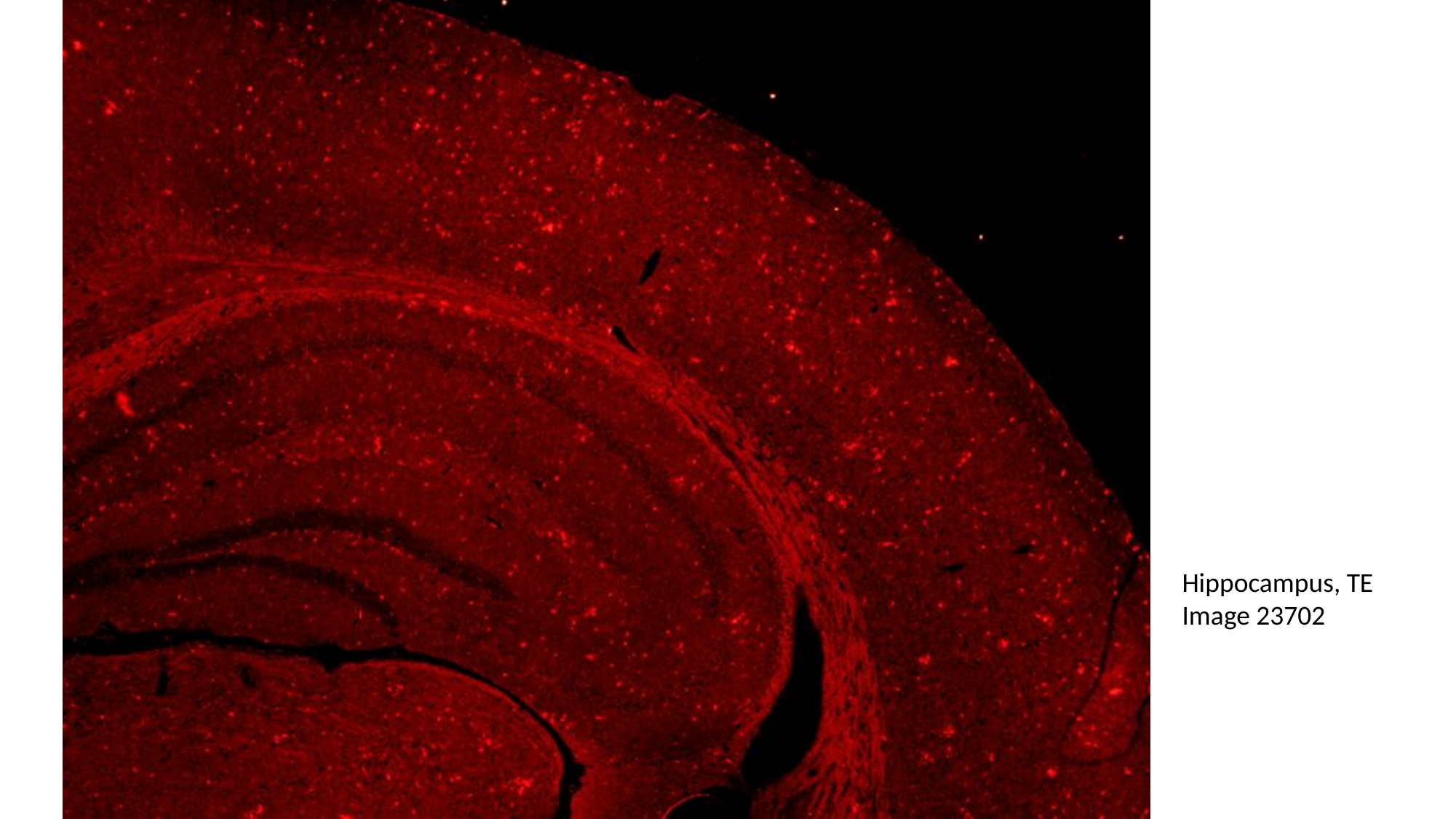

Hippocampus, TE
Image 23702

## Slide 8
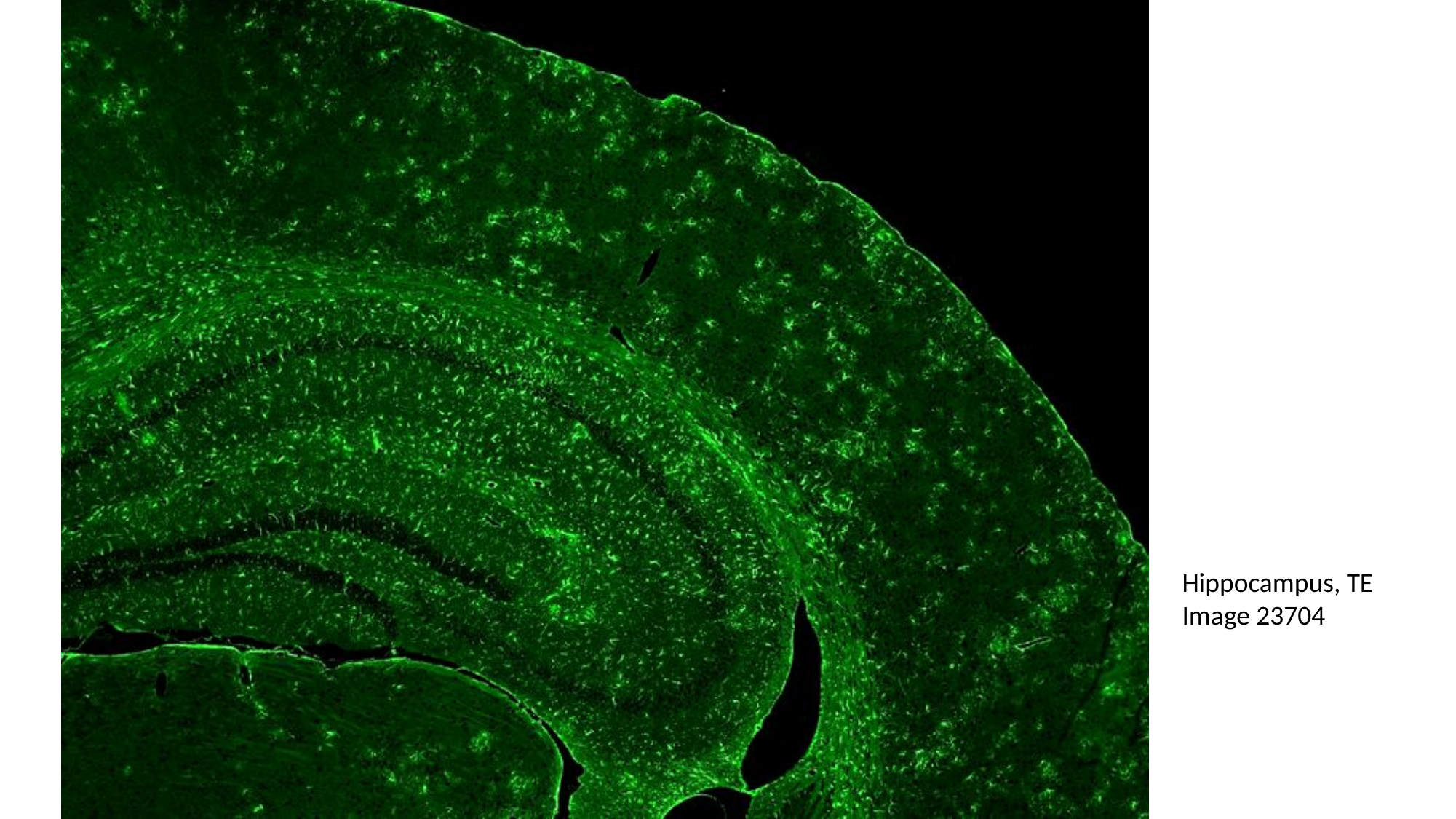

Hippocampus, TE
Image 23704

## Slide 9
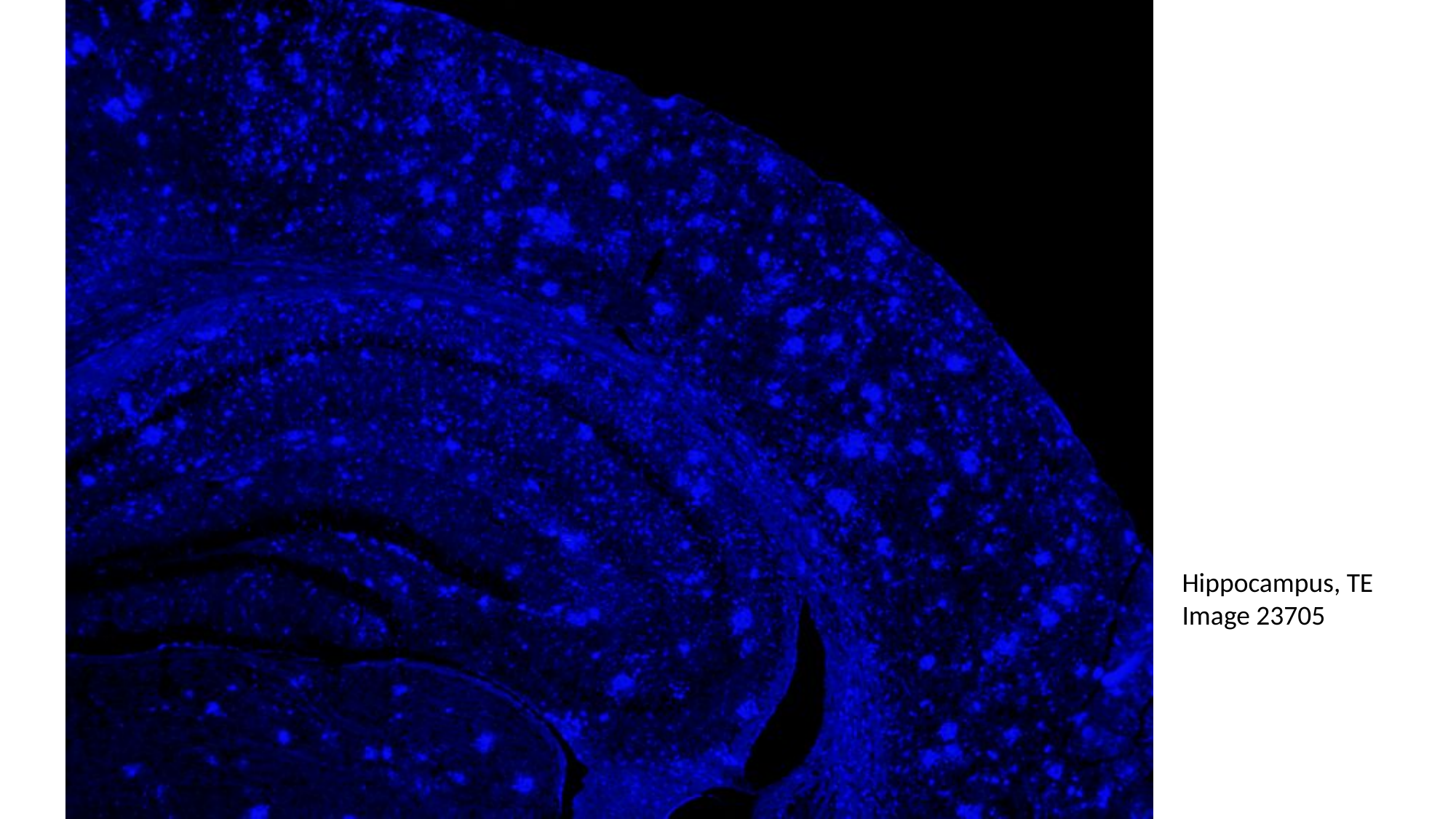

Hippocampus, TE
Image 23705

## Slide 10
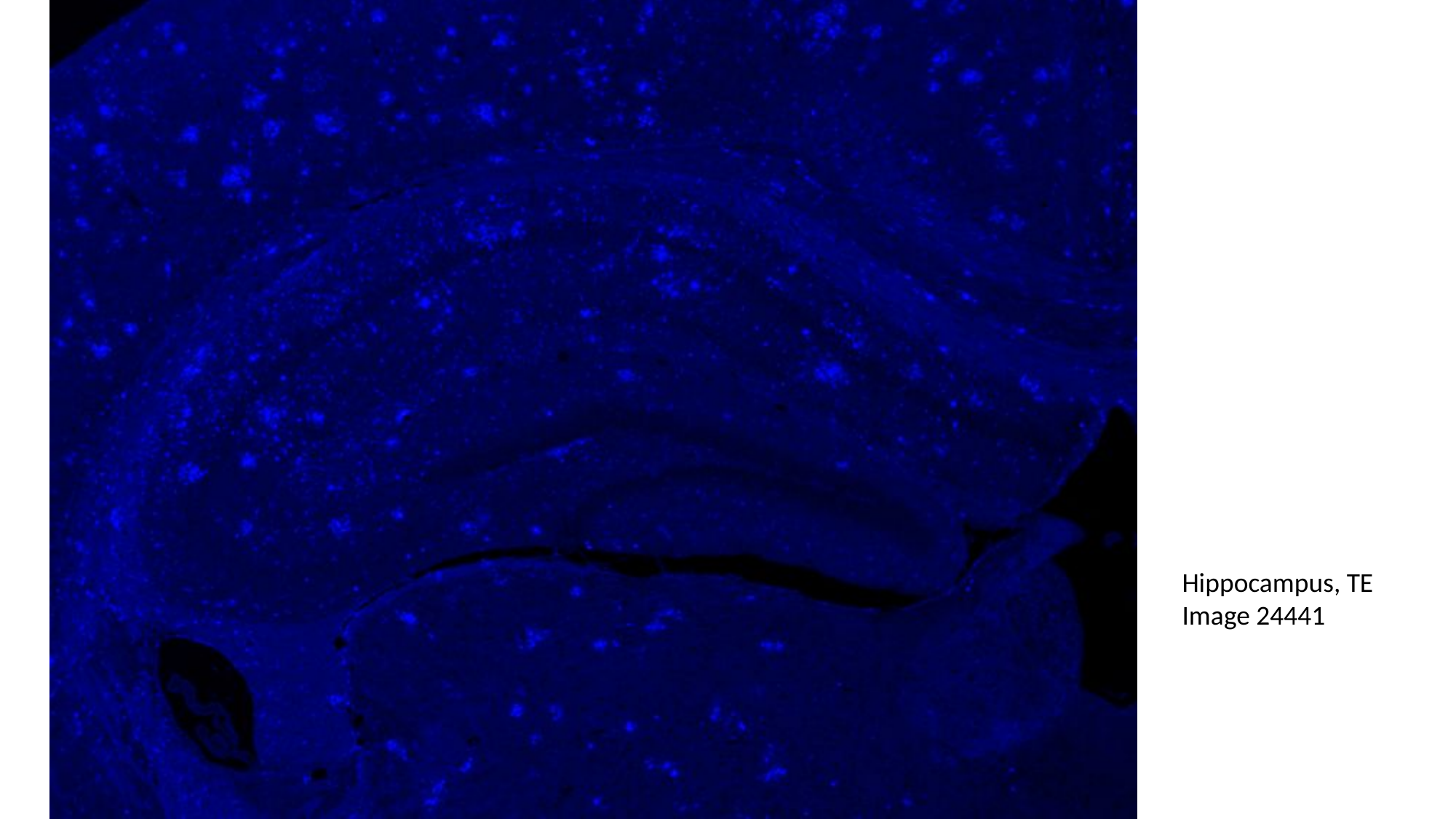

Hippocampus, TE
Image 24441

## Slide 11
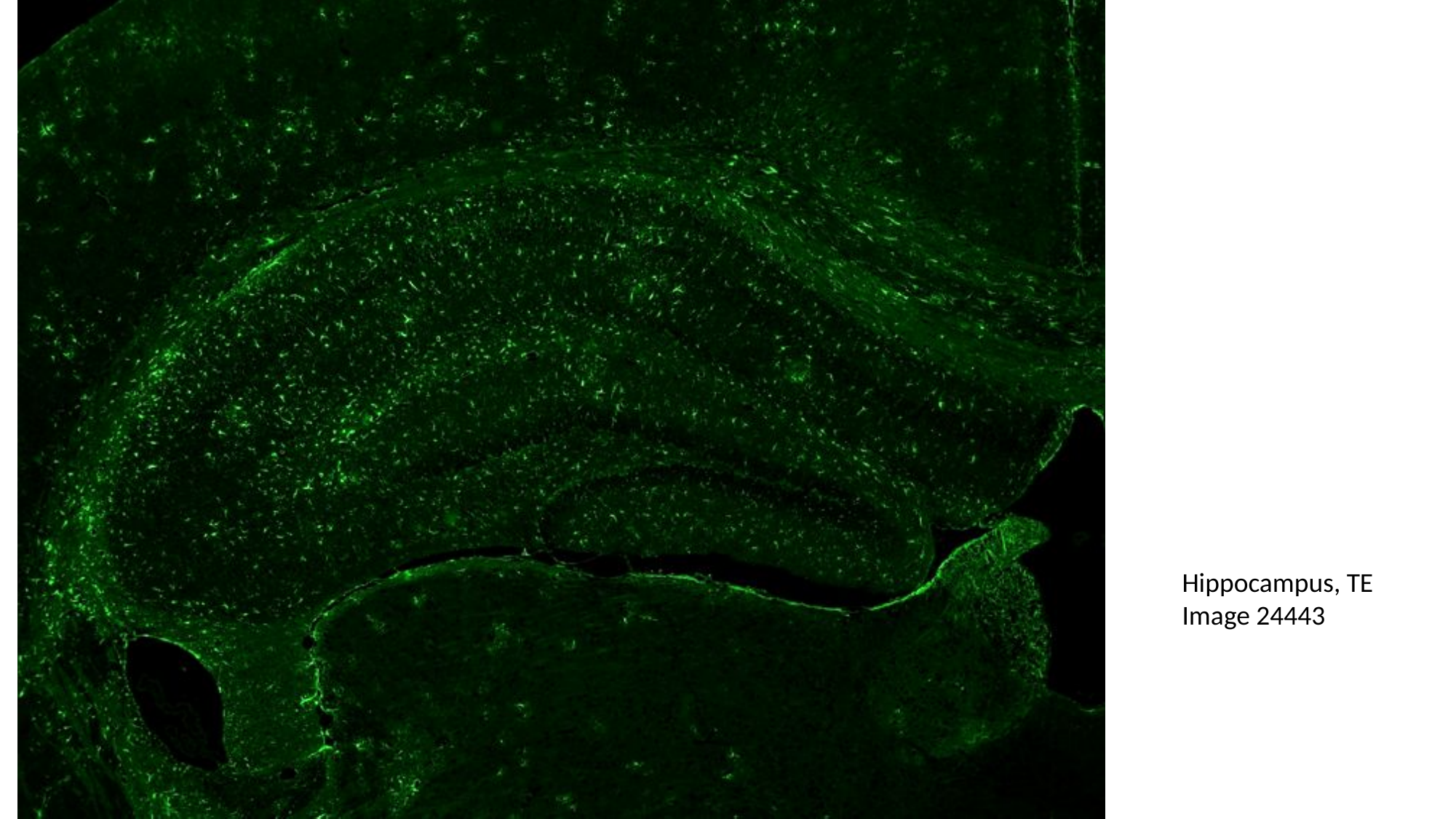

Hippocampus, TE
Image 24443

## Slide 12
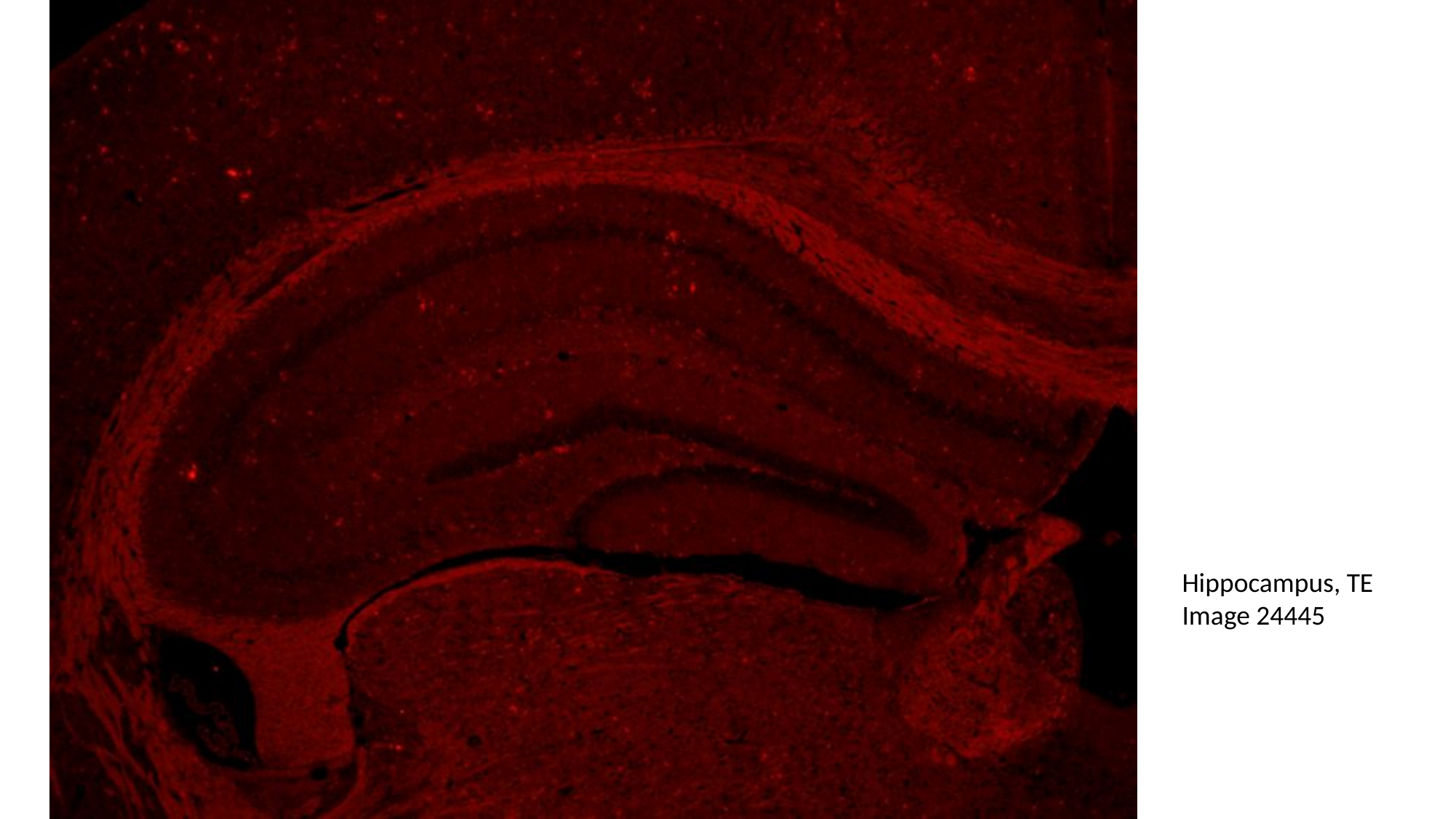

Hippocampus, TE
Image 24445
