# Supplementary figures and images for: Effects of early tooth loss on chronic stress and progression of neuropathogenesis of Alzheimer’s disease in adult Alzheimer’s model AppNL-G-F mice
Source: Front Aging Neurosci. 2024 Feb 26;16:1361847. doi: 10.3389/fnagi.2024.1361847 (PMC10925668; doi:10.3389/fnagi.2024.1361847)

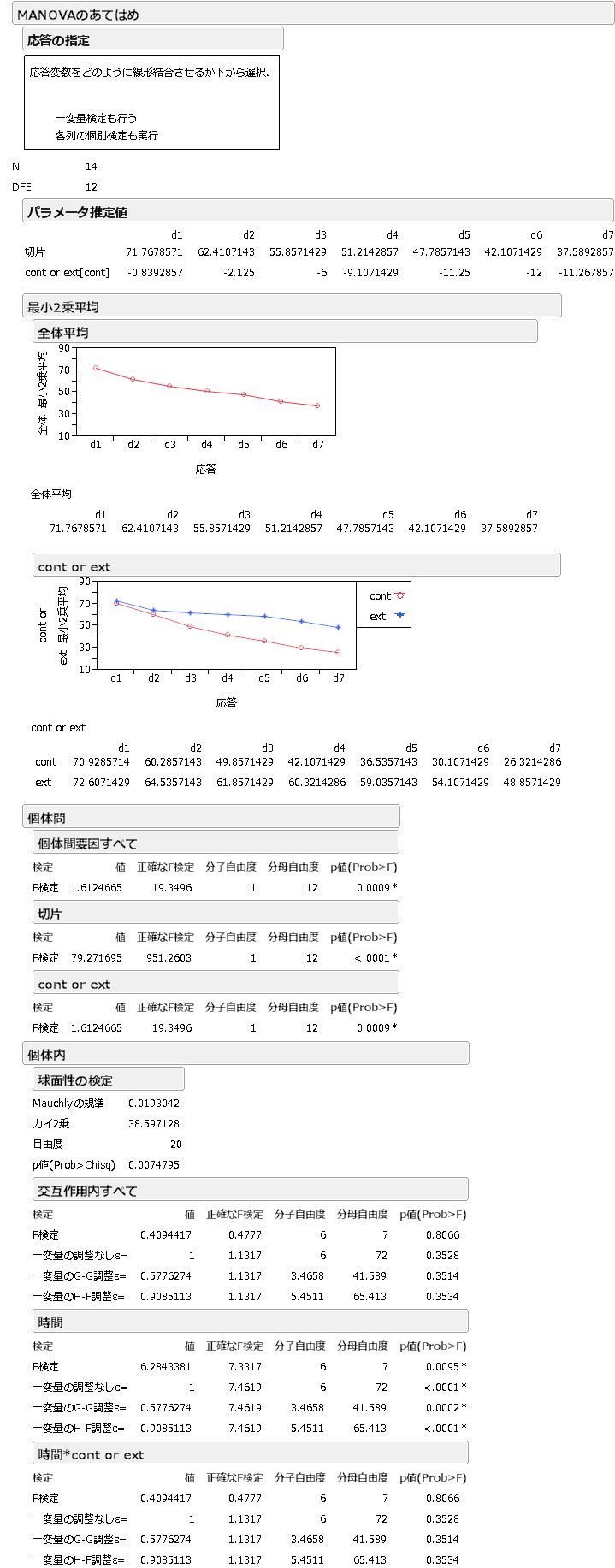

Supplement: Supplementary file 6 [file Image_1.JPEG]
